# Revealing role of epigenetic modifiers and DNA oxidation in cell-autonomous regulation of Cancer stem cells

**DOI:** 10.1186/s12964-024-01512-1

**Published:** 2024-02-12

**Authors:** Alejandra I. Ferrer-Diaz, Garima Sinha, Andrew Petryna, Ruth Gonzalez-Bermejo, Yannick Kenfack, Oluwadamilola Adetayo, Shyam A. Patel, Anupama Hooda-Nehra, Pranela Rameshwar

**Affiliations:** 1 Department of Medicine – Division of Hematology/Oncology, Rutgers, New Jersey Medical School, Newark, NJ 07103 USA; 2 Rutgers School of Graduate Studies at New Jersey Medical School, Newark, NJ USA; 3 University of Puerto Rico, Cayey, PR Puerto Rico; 4 Rutgers School of Dental Medicine, Newark, NJ USA; 5 Division of Hematology and Oncology, Department of Medicine, UMass Memorial Medical Center, UMass Chan Medical School, Worcester, MA USA; 6 Rutgers Cancer Institute of New Jersey, Newark, NJ USA

**Keywords:** Breast cancer, Dormancy, Epigenome, Resistance, Breast cancer, Cancer stem cell

## Abstract

**Background:**

Breast cancer cells (BCCs) can remain undetected for decades in dormancy. These quiescent cells are similar to cancer stem cells (CSCs); hence their ability to initiate tertiary metastasis. Dormancy can be regulated by components of the tissue microenvironment such as bone marrow mesenchymal stem cells (MSCs) that release exosomes to dedifferentiate BCCs into CSCs. The exosomes cargo includes histone 3, lysine 4 (H3K4) methyltransferases - KMT2B and KMT2D. A less studied mechanism of CSC maintenance is the process of cell-autonomous regulation, leading us to examine the roles for KMT2B and KMT2D in sustaining CSCs, and their potential as drug targets.

**Methods:**

Use of pharmacological inhibitor of H3K4 (WDR5–0103), knockdown (KD) of KMT2B or KMT2D in BCCs, real time PCR, western blot, response to chemotherapy, RNA-seq, and flow cytometry for circulating markers of CSCs and DNA hydroxylases in BC patients. In vivo studies using a dormancy model studied the effects of KMT2B/D to chemotherapy.

**Results:**

H3K4 methyltransferases sustain cell autonomous regulation of CSCs, impart chemoresistance, maintain cycling quiescence, and reduce migration and proliferation of BCCs. In vivo studies validated KMT2’s role in dormancy and identified these genes as potential drug targets. DNA methylase (DNMT), predicted within a network with KMT2 to regulate CSCs, was determined to sustain circulating CSC-like in the blood of patients.

**Conclusion:**

H3K4 methyltransferases and DNA methylation mediate cell autonomous regulation to sustain CSC. The findings provide crucial insights into epigenetic regulatory mechanisms underlying BC dormancy with KMT2B and KMT2D as potential therapeutic targets, along with standard care. Stem cell and epigenetic markers in circulating BCCs could monitor treatment response and this could be significant for long BC remission to partly address health disparity.

**Supplementary Information:**

The online version contains supplementary material available at 10.1186/s12964-024-01512-1.

## Introduction

Breast cancer (BC), the most common cancer in women, remains a clinical problem [1]. A major clinical issue is linked to challenges to target dormant cancer cells [2]. The preference of BC for bone marrow (BM) results in poor prognosis [3]. Entry of BC cells (BCCs) in the BM occurs at any time during the disease as well as the period before clinical diagnosis. The latter could be years to decades when the cancer cells remain dormant [4, 5]. In BM, BCCs can survive as dormant cells for long- periods, even decades [6]. Dormant BCCs remain in cycling quiescence, resist treatment, and adapt properties of stem cells [7–10]. These properties have led to dormant BCCs referred as cancer stem cells (CSCs) [9, 11]. The stem cells properties of dormant BCCs are in line with the ability of these cells to reactivate into tertiary metastasis [9, 12].

Drug resistance of dormant BCCs involves a complex mechanism. In addition, one must consider that developing methods to treat dormant BCCs could be influenced by shared properties with healthy endogenous stem cells. As an example, in BM, dormant BCCs and hematopoietic stem cells (HSCs) share anatomical location. The makes it difficult to target the dormant cells without untoward effects on HSCs [13, 14]. In this regard, imperative survival of HSCs would limit the therapeutic dose of a drug that targets dormant BCCs. Thus, it is important to understand how BCCs achieve dormancy in BM since this would allow for strategic development of methods to safely eradicate BCCs without affecting the hematopoietic system.

Cells within tissue environment could influence BC dormancy, partly by altering the cells’ epigenome leading to dedifferentiation into CSCs [13, 15–18]. BM endogenous cells such as macrophages, fibroblasts and mesenchymal stem cells (MSCs) support of BC dormancy [5, 19, 20]. BCCs can undergo dedifferentiation to CSCs after instructing MSCs in BM to release exosomes with distinct mRNA cargo [18, 19]. Including among the exosomal cargo are transcripts for DNA and histone modifiers - DNA methyltransferase-1 (DNMT1), and histone 3, lysine 4 (H3K4) methyltransferases - KMT2B and KMT2D.

A focus on epigenetic modifiers in cancer, including the present study on BC dormancy is based on the diminished paradigm of genomic instability as the sole contributor to cancer development [21, 22]. Low levels of gene mutations in some cancers strongly support roles for epigenomic modification, including functions associated with BC [23–25]. There are six members of the lysine methyltransferase 2 (KMT2) family of proteins, formerly referred to as mixed-lineage leukemia (MLL). KMT2 can incorporate up to three methyl group(s) at lysine 4 residues of histone 3 tail ends, resulting in epigenetic marks that signals transcriptional activation [26, 27].

KMT2B and KMT2D, H3K4 methyltransferases, are involved in human development [28]. Abnormal placement of H3K4 methylation marks across the genome has been associated with cancer and patient survival [29]. Reduced H3K4me2 is linked to poor survival whereas decreased H3K4me3 could significantly improve patient prognosis [29]. Although H3K4 methylation is associated with transcriptional activation, the overall cellular response depends on the deposited marks, and the affected downstream activated genes [29]. Mutation of KMT2B in cancers frequently leads to upregulated levels [30]. In BC, KMT2B is recruited by the estrogen receptor-α to the IL-20 promoter to enhance H3K4 methylation and BCC proliferation [31]. Targeting of both KMT2B and IL-20 in BC disrupts estrogen signaling [31]. In a pan-cancer study evaluating the -intra and -inter tumor heterogeneity of the KMT2 gene, it was noted that KMT2B expression was significantly upregulated in 18 tumor types, including BC subtypes, which correlated with an aggressive phenotype [32]. Mutation of KMT2D in BC results in treatment resistance [33, 34]. Inhibition of KMT2D with current treatments such as PI3K inhibitors reduced tumor volume in ER-positive BC [35, 36]. While KMT2D is oncogenic, its subunit, UTX, can function as a tumor suppressor, reducing epithelial-to-mesenchymal transition (EMT) [37].

DNA methylation is a heritable epigenetic mark that dictates cellular identity by regulating gene expression [38]. Deposition of a methyl group on the fifth carbon of cytosines (5-methylcytosine, 5mC) is a conserved epigenetic modification implicated in cellular memory and differentiation [39]. 5mC modifications are specific to palindromic sequences of cytosines linked to guanines by phosphodiester bonds (CpG islands) [38, 40]. DNA methylation patterns are catalyzed by the DNA methyltransferase (DNMT) family - DNMT1, DNMT3A, and DNMT3B. DNMT1 is involved in the maintenance of methylated cytosines and acts preferably on hemimethylated DNA [41]. Conversely, de novo methylation is accomplished by DNMT3A/B in a symmetric manner [42]. Aberrant DNA methylation patterns are involved during the development and progression of cancer. DNMT1 expression is upregulated in BC tumors, and its deletion impedes CSC self-renewal and survival [43]. The degree of DNMT1 expression depends on the BC subtype. For instance, triple negative BC (TNBC) and inflammatory BC have higher expression of DNMT1 as compared to luminal A BC [44]. DNMT1 reduces expression of estrogen receptor, promotes EMT and allows for expansion of the CSCs in TNBC [45–47].

BCCs can enter and exit dormancy depending on cues from the microenvironment. Dedifferentiated BCCs can adapt long-term dormancy by forming gap junctional intercellular communication (GJIC) with BM resident hematopoietic and non-hematopoietic cells [18, 19, 48]. BM microenvironmental cells can alter cytokine production and other secretomje by BCCs [20, 49]. MSC-derived exosomes induced BCC dedifferentiation to CSCs [18]. Recapitulating the hematopoietic matrix with 3D bioprinting containing printed BCCs indicated BC dedifferentiation by cell-autonomous mechanisms [50]. CSCs in the BM have survival advantages – cycling quiescence by forming GJIC with the endogenous stromal cells, and using MSCs to protect them from immune elimination [51, 52]. Thus, it is critical to understand how CSCs survive. Since the method of cell autonomous dedifferentian of BCCs remains poorly studied, we studied the role of two *KMT2* genes in this process, using in vitro and in vivo studies. This study reports on parallel studies with circulating BC from patients for stem cell markers, DNMT associated hydroxylase. We discussed the findings in the context of patients’ treatment and diagnoses, as well as the combined roles for DNA methylation by DNMT and H3K4 in sustained dormancy.

## Materials

### Ethics statement

#### Human subjects

Rutgers Institutional Review Board (IRB) approved the use of blood from BC patients (Table 1).

**Table 1:** Patient demographics

Patients	Age (Yrs)	Stage (Diagnosis)	Hormone Status	Treatment
1	40	3	Her2+ ER-	Pertuzumab, Trastuzumad Docetaxel, carboplatin
2	75	3a	Her2- ER+	no treatment
3	50	4/Relapse	Her2- ER-	Abraxane, Gemcytabine
4	65	2a	Her2+ ER+	Pertuzumab, Transtuzumab*
5	64	4/relapse	Her2- ER-	Sacituzumab, Govitecan
6	89	2a	Her2- ER+	Exemestane
7	67	4/relapse	Her2- ER+ (minimal)	Sacituzumab, Govitecan
8	55	3a	Her2- ER+	Anastrozole **
9***	64	2	Her2+ ER+	Pertuzumab, trastuzumab, carboplatin, docetaxel
10***	45	4	ER- Her2-	Adriamycin, Cyclophosphamide

*Post-treatment (ended 6/23), followed by endocrine therapy (Letrozol). At relapse, treated with Docetaxel and Carboplatin. Due to low tolerance, switched to listed treatment (Patient 4)

** Patient was treated between July 2020–November 2020. This followed surgery. At blood draw, patient was on endocrine therapy with Anastrozole. Currently no active disease

***Patients 9 and 10 blood samples were analyzed with in vitro studies (Table 3)

#### Mice

The use of nude mice was approved by Rutgers Institutional Animal Care and Use Committee (IACUC), Newark Campus. Rutgers IACUC is accredited by the Association for Assessment and Accreditation of Laboratory Animal Care (AAALAC). Mice were housed in the Comparative Medicine Resource center at Rutgers New Jersey Medical School, Newark, NJ.

### Reagents

DMEM, RPMI-1640, L-glutamine, penicillin, streptomycin, dimethyl sulfoxide, geneticin, Glycerol, optiMEM, polybrene, trizol, trypan blue stain, platinum SYBR Green qPCR Supermix-UDG Kit, Supersignal West Femto Maximum Sensitivity Substrate, High-Capacity cDNA Reverse Transcription kit, and Total exosome isolation reagent were purchased from Thermo Fisher Scientific (Waltham, MA); ammonium persulfate, bovine serum albumin, magnesium chloride, N,N,N′,N′-Tetramethylethylenediamine (TEMED), NP-40, EDTA-free protease inhibitor, sodium chloride, fetal bovine sera (FBS), Ficoll Hypaque and Triton-X100 from Millipore-Sigma (St. Louis, MO); protein loading dye, Bradford protein reagent, and sodium dodecyl sulfate from BioRad (Hercules, CA). WDR5–0103 and doxorubicin were purchased from Tocris Bioscience (Minneapolis, MN). Acryl/Bis Solution (30%) 37.5:1 was purchased from VWR (Radnor, PA); puromycin from InvivoGen (San Diego, CA); TransIT-Lenti transfection reagent from Mirus; MTT-Assay and 5-Aza-2′-deoxycytidine from Abcam (Waltham, MA); p24 Rapid Titer Kit from Takara Bio (Mountainview, CA); and plasmid miniprep kit and RNeasy Mini Kit from Qiagen (Germantown, MD). Carboplatin and doxorubicin were obtained from University Hospital Pharmacy (Newark, NJ).

### Antibodies

All primary human antibodies were against human antigens. Rabbit anti-cyclin D1 (1:1000 dilution), rabbit anti-Ki67 (1:250 dilution), and rabbit anti-vinculin (1:1000 dilution) were purchased from Abcam (Waltham, MA); rabbit anti-human p38 (1:1000 dilution), rabbit anti-MDR1 (1:1000 dilution), rabbit anti-CDK4 (1:1000 dilution), mouse anti-EpCam-AlexaFluor488 (1:100 dilution), goat anti-Tet2 (1:200 dilution), rabbit anti-5-hmC (1:200 dilution), and rabbit anti-human CDK6 (1:1000 dilution) from Cell Signaling (Danvers, MA); goat anti-rabbit IgG (1:2000 dilution) from ThermoFisher Scientific; goat anti-rabbit Alexa Fluor 594 (1:500 dilution) from Invitrogen (ThermoFisher); mouse anti-pan cytokeratin-PE (1:200 dilution), mouse anti-Oct 3/4-PerCPCy5.5 (1:5 dilution) from Becton Dickinson (San Jose, CA); donkey anti-rabbit IgG-AlexaFluor647 and donkey anti-goat IgG-AlexaFluor488 from Life Technologies; BD Lysing solution (Becton Dickinson).

### Vectors

pOct4a-GFP was donated by Dr. Wei Cui (Imperial College, London, UK) and was previously described from studies by our group [8, 9, 18, 48]. The description of pOct4a-dsRed was previously described [8]. Human KMT2B, KMT2D shRNA clones, and scramble sequence shRNA for psi-LCRU6GP were purchased from GeneCopoiea (Rockville, MD).

### Cell lines

Triple negative MDA-MB-231 and triple positive T47D BCCs were obtained from American Type Culture Collection and cultured as per their instruction. Cells were grown in DMEM (MDA-MB-231) and RPMI supplemented with insulin (T47D). Both type of media contained 10% FBS, 2 mM L-glutamine, 100 IU/ml penicillin, 100 μg/ml streptomycin and 1% non-essential amino acid. HEK293T cells were cultured in DMEM with 10% FCS.

MDA-MB-231 cells were stably transfected with pOct4a-GFP or pOct4a-dsRED, as described [9]. Fluorescence intensities correlated with the expression of the stem cell gene, Oct4a [9]. We selected and maintained cells expressing the reporter genes with Geneticin (500 μg/ml). Previous studies showed BCCs expressing with the highest fluorescence (top 5%) were CSCs [9].

### Treatment of BCCs with H3K4 inhibitor WDR5–0103

BCCs were seeded in 6-well plates at 3.5 × 10^5^ cells/well. After overnight incubation, the cells were treated with WDR5–0103 or vehicle. The media were replaced after 24 h. At 48 h, BCCs were de-adhered with 0.25% trypsin-EDTA and then subjected to the following readouts: cell viability, flow cytometry and real time PCR.

### Preparation of lentiviral particles

Lentiviral particles were prepared as described [48]. The shRNA viral plasmids were inserted into One Shot Mach1T1 Phage-Resistant (Thermo Fisher Scientific) chemically competent *E. coli*. The DNA from the transformed bacteria was isolated with Plasmid Miniprep Kit (Qiagen). The DNA was digested with *BamH1* and *EcoRI* followed by gel electrophoresis validation. The transformed bacteria were cultured and amplified in luria broth containing ampicillin (50 μg/ml). The plasmid DNA was collected for isolation of lentiviral particles. The isolated plasmids were propagated in HEK-293 T cells (90% confluence). The packaging plasmids (5 μg/ml) were gently combined with the lentiviral plasmid of interest (5 μg/ml). The mixture was transferred to a tube containing 1 ml of Opti-MEM for gentle mixing, followed by adding 30 μl of TransIT-Lenti (Mirus Bio) reagent. The mixture was homogenized and then incubated at room temperature for 10 min to allow for the formation of transfection complexes. The mix was added dropwise to HEK-293 T cells followed by incubation at 37 °C for 48 h. The media were collected and centrifuged at 300 *g* for 10 mins to remove the cellular debris. Supernatant containing the virus was filtered through 0.45 μm PVDF membrane and concentrated using the Lenti-X Concentrator (Takara Bio). The concentrated viral clones of KMT2B, KMT2D, DNMT1, and scramble shRNA were quantified with the Lenti-X p24 Rapid Titer Kit (Takara Bio). The viral nix was aliquoted into 0.5 ml low-protein binding tubes and stored at − 80 °C.

### Knockdown (KD) of KMT2B and KMT2D

MDA-MB-231 and T47D BCCs with stable pOct4a-dsRed were seeded at 5 × 10^4^/well in 24-well plates. We selected pOct4a-dsRed because the lentivirus for KMT2B and KMT2D shRNA express GFP (Figs. S1 and S2). After 24 h, the cells were transduced with KMT2B, KMT2D shRNA, or scramble shRNA at a multiplicity of infection of 1:1 and 4 μg/ml of polybrene. After 48 h, the media were replaced with fresh media supplemented with 1.5 μg/ml of puromycin every 2 days for 2 weeks. The efficiency of transduction was evaluated by fluorescence microscopy for GFP and western blot for the appropriate protein linked to the shRNA (Figs. S1 and S2).

### Carboplatin treatment and cell viability

Trypan blue staining was used to assess cell viability in cultures with BCCs treated with WDR5–0103 or vehicle. Similar assessments were conducted for BCCs with KMT2B or KMT2D KD, or scramble sequence. Cells were manually counted on a hemocytometer.

BCCs were seeded at 2 × 10^4^ cells/well in a 96-well plate. After overnight incubation, the cells were treated with vehicle or carboplatin (220 μg/ml) every 24 h or 2 days. After this, the cells were subjected to the MTT assay (Abcam) as per manufacturer’s instruction. Briefly, media were removed and replaced with the MTT reagent, diluted in serum-free media. The cells were incubated at 37° for 3 h. This was followed by adding MTT solvent to neutralize the reaction. The cells were incubated on a shaker for 15 mins at room temperature and then analyzed by measuring the absorbance at 590 nm on the Synergy HTX (Biotek) microplate reader.

## Flow cytometry

### GFP and dsRED

Flow cytometry analysis was conducted to determine Oct4a GFP/dsRed intensity in BCCs after treatment with the epigenetic inhibitors and silencing of the epigenetic mediators. Cells were collected, resuspended in 500 μl of 1X PBS, and placed in 12x75mm polystyrene tubes (MTC Bio). The cells were analyzed on a FACS Calibur (BD Biosciences) for GFP/dsRed intensities. The data were analyzed with FlowJo software (BD Biosciences). We designated the relative maturity of BCCs based on our previous reports [9]. Cells within the top 5% of GFP/dsRed fluorescence were designated as long-term repopulating CSCs (Oct4a-GFP/dsRed^hi^). This was followed by Oct4aGFP/dsRed^med^, Oct4aGFP/dsRed^low^ (early progenitors), and Oct4aGFP/ dsRed^neg^ (late progenitors).

### Phenotype of circulating BCCs in blood

Red blood cells were lysed with BD FACS lysing solution, as per manufacturer’s instructions. The lysed cells were tested with two panels of antibodies: Panel 1 contained antibodies against pan cytokeratin, Oct3/4 and EpCam; Panel 2 contained pan cytokeratin, Oct3/4, Tet2 and 5hmC. The concentrations of the antibodies are listed above. The concentrations of isotype added to the cells were similar to the amount of test antibodies.

Panel 1 used intracellular labeling by permeabilization at 4 °C with 0.1% Triton X-100 for 10 mins. Cells were washed with 1x PBS and then labeled with the test antibodies and isotype controls. The tubes were incubated in the dark for 30 mins at 4 °C followed by washing with 1x PBS. The cells were immediately analyzed on the FAC Calibur.

Panel 2 labeling used intracellular and extracellular labeling. The latter was first done by fixing with 3.7% formaldehyde at room temperature for 15 mins. Cells were washed with 1x PBS and then labeled with anti-EpCam and isotype control. The incubation and wash were performed as for intracellular labeling. After this, the cells were labeled intracellularly for pan-cytokeratin, Oct3/4, Tet2 and 5hmC as for Panel 1.

## Treatment of peripheral blood mononuclear cells (PBMCs) from BC patients

Due to limited blood supplies, we studied the last two patients (#s 9 an 10, Table 1) for in vitro response to chemotherapy and the DNA methylation inhibitor, Azacitidine. PBMCs were isolated from the patients’ blood by Ficoll Hypaque gradient centrifugation. Cells (5 × 10^6^) were added to 2 mL RPMI 1640 with 10% FBS. The cultures were incubated with vehicle (PBS), carboplatin (200 μM) alone, or with Azacitidine (2 μM). At day 7, the cells were analyzed by flow cytometry using Panels 1 and 2 antibodies, as described above for flow cytometry.

## Real-time PCR

Total RNA was isolated using TRIzol reagent according to the manufacturer’s instruction (Thermo Fisher Scientific). The RNA was reverse transcribed to cDNA with the High-Capacity cDNA Reverse Transcription kit (Thermo Fisher Scientific) and amplified using the GeneAmp PCR System 9700 (Applied Biosystems). The cDNA was diluted with nuclease-free water to 200 ng/μl and then mixed with SYBR Green PCR Master Mix, primers of interest, and nuclease-free water. Real-time PCR was conducted on a 7300 Real-time PCR system (Thermo Fisher Scientific) at 50 °C for 2 mins, 95 °C for 10 mins followed by 40 cycles of 95 °C for 15 seconds and 60 °C for 1 minute. The following primers were used in the PCR mix: Oct4a: Forward 5’ctg aag cag aag agg atc ac 3′; Reverse 5’gct ttg cat atc tcc tga ag 3′; KLF4: Forward 5’aac ctt acc act gtg act gg 3′; Reverse 5’cat atc cac tgt ctg gga tt 3′; Nanog: Forward 5′ caa tgg tgt gac gca ggg at 3′; Reverse 5′ gac tgg atg ttc tgg gtc tgg 3′; Notch1: Forward 5’cca agt ata gcc tat ggc aga a 3′; Reverse 5′ aag tct gac gtc cct cac 3′; Sox2: Forward 5’taa ctg tcc atg cgc tgg tt 3′; Reverse 5′ agg atat agt aca cgc tgc cc 3′; β-actin: Forward 5’gcc cta taa aac cca gcg gc 3′, Reverse 5’aga ggc gta cag gga tag ca 3′; GAPDH: Forward 5′ cag aag act gtg gat ggc c 3′, Reverse: 5′ cca cct tct tga tgt cat c 3′.

The PCR data were analyzed by calculating the expression values of the experimental samples, relative to the housekeeping control using the following method: (experimental sample value = 2^(average of housekeeping gene value-gene of interest value)^. The PCR readouts that were 40 or higher were considered as zero and not assessed for differential expression. The fold change in gene expression was calculated by first dividing the mean of technical experimental replicates by the mean of the technical control. The control biological replicates were then normalized to 1 to determine the fold change of the biological experimental replicates.

## Western blot

Cells were resuspended in 50–100 μl of lysis buffer, which contained 50 mM Tris-HCL (pH 7.4), 100 mM NaCl, 2 mM MgCl_2_, 10% glycerol, 1% NP-40, and two tablets of EDTA-protease inhibitor cocktail (Millipore-Sigma). Cell lysates were subjected to freeze/thaw cycles - 2 mins in liquid nitrogen followed by 2 mins in a 37 °C water bath. Cell lysates were centrifuged at 2000 *g* for 10 mins and the supernatants containing the proteins were collected and then assessed for protein levels with the Bradford Protein Assay Reagent (BioRad). The analyses used BSA (BioRad) to establish a standard curve. Extracts (15 μg) were electrophoresed on 12% SDS-PAGE gel and then transferred onto Immobilon-P PVDF membranes (ThermoFisher Scientific). The membranes were washed with 1X PBS-tween for 10 mins and then blocked for 20 mins with 3% non-fat milk diluted in 1x PBS. The membranes were incubated overnight at 4 °C on a shaker with the primary antibodies - anti-p38, anti-CDK4, anti-CDK6, anti-cyclinD1, anti-MDR1, anti-KMT2B, anti-β-actin, or anti-vinculin at a 1:1000 in 3% non-fat milk. Membranes were washed and blocked with 3% non-fat milk, followed by incubation with HRP tagged secondary antibodies at 1:2000 in 3% non-fat milk for 2 h at 4 °C. The membranes were washed for 20 mins and then developed with the Super Signal West Femto Maximum Sensitivity Substrate for 5 mins. The protein bands were imaged using the using the ChemiDoc XRA (BioRad).

## Scratch assay

BCCs were seeded at 3 × 10^5^ cells/well in 6-well plates. Once the cells achieved 100% confluency, a scratch was performed in the middle of the well from top to bottom using a 200 μl pipette tip. Gap closure was assessed at 0, 24, 48, 72, and 96 h using the EVOS Fl Auto 2 imager.

## RNA sequencing (RNA-seq)

Total RNA was extracted with RNAeasy mini kit (Qiagen) from KMT2B, KMT2D KD MDA-MB-231, or with scramble shRNA. The samples were submitted to the Genomics Center at Rutgers New Jersey Medical School for RNA-seq. Depletion of ribosomal RNA was conducted with the Ribo-Zero Gold kit (Illumina) followed by preparation of Next-Generation sequencing cDNA libraries using the NEB Ultra II Library Preparation Kit and NEBNext Multiplex Oligos for Illumina (Dual Index Primers Set 1). Quality control of the libraries was assessed with the Qubit high sensitivity kit and fluorometer (Thermo Fisher Scientific), Tapestation 2200 instrument and D1000 ScreenTapes (Agilent). Next, the cDNA libraries were diluted to 2 nM, denatured, and sequenced on the NextSeq instrument using the 1X75 cycle high throughput kit. The BCL output files from the sequencing machine were converted into FASTq files with the BCL2FASTQ software (Illumina).

## Data analyses

Normalization of RNA-seq data and identification of differentially expressed genes was assessed with the EdgeR package from R by using the Galaxy software. Genes with a fold change of − 1.5 to + 1.5 and a *p*-value< 0.05 were considered as differentially expressed. The data were visualized through generation of principal component analyses (PCA) plot, heatmap, and volcano plot with an adjusted *P*-value of 0.05 as cutoff. Gene set enrichment analysis was conducted with the Hallmark Gene set database. Identification and analysis of significant cellular pathways from the dataset was performed with the Ingenuity Pathway Analysis (IPA) software (Qiagen). A pathway was regarded as significant by the following cutoffs: genes exhibited a *p*-value< 0.05, expression log ratio of 1, and activated z-score of 2.

### In vivo BC dormancy

#### Intravenous route

The method to establish BC dormancy in nude mice was previously described [9, 19]. BCCs, knockdown for KMT2B or KMT2D, or transduced with scramble shRNA (5x10^5^cells in 300 μl) were injected intravenously into 6-wk old nude athymic female mice. On days 3 and 5, mice were injected intraperitoneally with a low dose of carboplatin (2 mg/kg) or vehicle to establish BC dormancy in the BM. The mice were euthanized on day 7 and the organs such as liver, brain, lungs, and femur were harvested and placed in 3.7% formaldehyde for 48 h. In addition, the endosteal region of one femur per mouse was scraped and imaged with the EVOS FL Auto 2 Imaging System for GFP+ BCCs. The harvested organs, including the other femur, were embedded in paraffin and sectioned at the Histology Core Facility at Rutgers New Jersey Medical School (Newark, NJ). The sections were labeled for BCCs by immunohistochemistry.

#### Orthotopic route

MDA-MB-231 BCCs with stable pOct4a-GFP (5 × 10^5^) were injected into the mammary fat pad of female (6 weeks) nude BALB/c. After 1 week, the mice were euthanized, and the femurs were harvested and scraped to evaluate the presence of BCCs.

### Immunohistochemistry

Paraffin-embedded tissue sections were incubated overnight at 56 °C. The sections were dewaxed with xylene and ethanol and rehydrated with deionized water. Antigen retrieval from the tissue sections was performed with citrate buffer for 30 mins in a pressured water bath. The slides were washed twice with 1X PBS for 5 mins, and the cells were permeabilized with 0.1% Triton X-100. Next, the sections were washed with 1X PBS, followed by adding-Ki-67, at a final dilution of 1:250. The slides were placed in a humidified chamber and incubated overnight at 37 °C. The following day, the tissue sections were washed thrice with 1X PBS. The slides were incubated for 2 h at room temperature with the secondary antibody at a final dilution of 1:500. The slides were washed and analyzed on the EVOS FL Auto 2.

### Statistical analyses

The data were analyzed on Sigma Plot 15 (Systat Software Inc) using the two-tail student’s t-test and two-way ANOVA to compare groups. A *p* value less than 0.05 was considered significant.

## Results

### Prediction of cell-autonomous mediated CSC maintenance

**Fig. 1:**
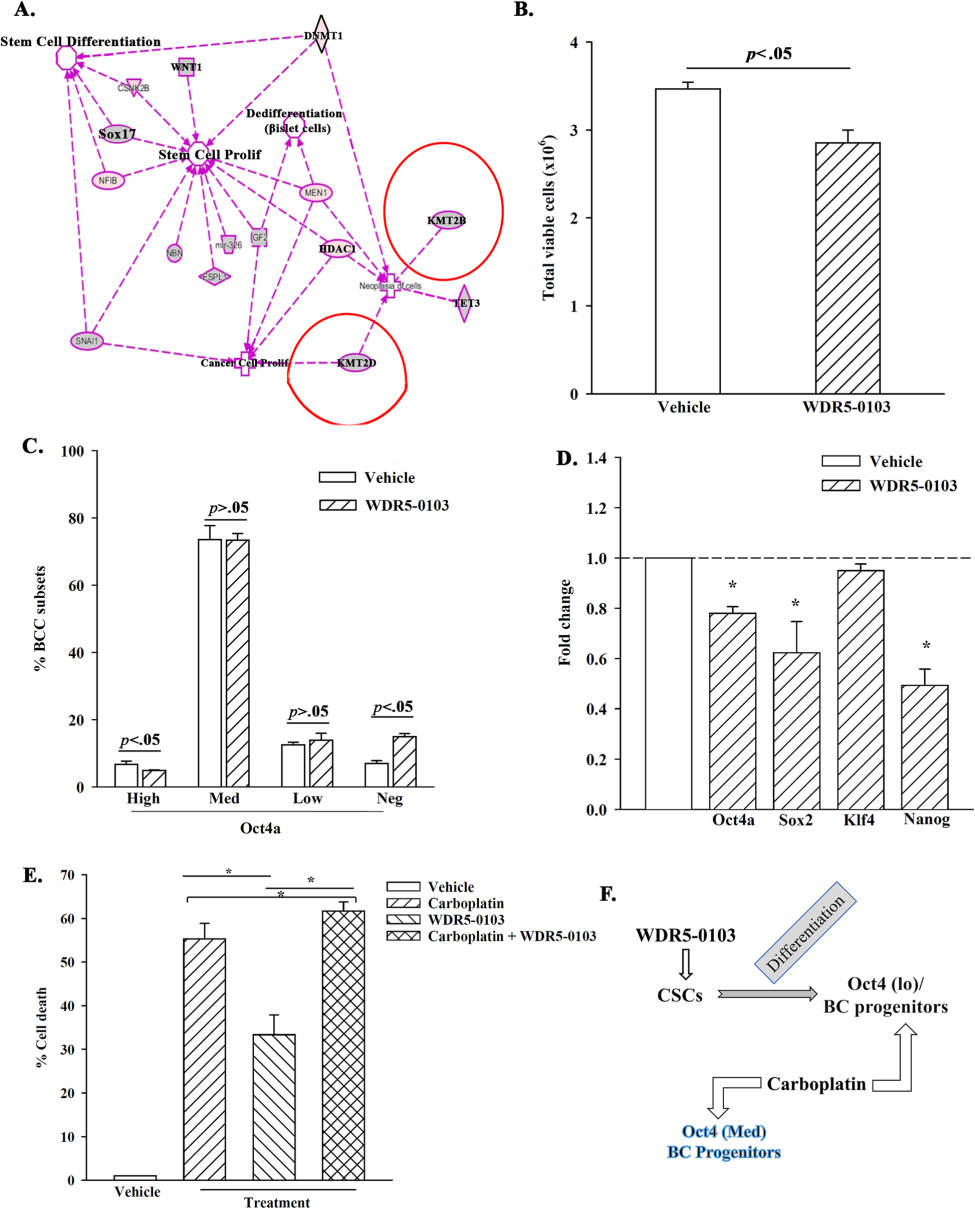
H3K4 as a potential target to differentiate CSCs. **A** Shown are the overlay of the epigenetic modifiers KMT2B, KMT2D, and DNMT1, with pathways associated with cancer development in IPA - differentiation of beta islet cells, proliferation of stem cells, proliferation of cancer cells, and neoplasia. The RNA-Seq data for the exosomes were previously reported [18]. **B** Viable MDA-MB-231 BCCs were counted after exposure to WDR5–0103 (10 μg/ml) using trypan blue exclusion. The data are presented for three biological replicates. **C** BCCs with WDR5–0103 (10 μg/ml) or vehicle. The viable cells were analyzed for BCC subsets by flow cytometry. Subsets were demarcated based on relative fluorescence intensities (Oct4a expression). The data are presented for four biological replicates. **D** Real time PCR assessed levels of the stem cell-associated genes - Oct4a, Sox2, Klf4, and Nanog in BCCs treated with WDR5–0103 or vehicle. The values for vehicle were assigned as 1 and the experimental values presented as fold change. The results are presented for three biological replicates. **E** MDA-MB-231 BCCs were treated with 10 μg/ml WDR5–0103 and/or 200 μg/mL carboplatin each day for 48 h. Control cells were treated with vehicle (DMSO). Cell death was assessed by trypan blue exclusion. The results are shown for three biological studies. *p < .05. **F** The diagram summarizes the findings in this figure – WDR5–0103 differentiates CSCs to Oct4(lo) BCC progenitors, known to cycle [18]. Addition of carboplatin target the Oct4(lo) and the non-CSCs

We previously reported on BCCs instructing MSCs to release exosomes with distinct RNA cargo to facilitate stepwise dedifferentiation of BCCs to CSCs [18]. BCCs induced changes within the MSC-released exosomal cargo to include transcripts for H3K4 modifiers, KMT2B and KMT2D. This led us to ask if endogenous KMT2B and KMT2D in CSCs can sustain multipotency. We first subjected the single cell RNA-seq data from the published studies to IPA [18]. In this study, the BCCs were treated with exosomes from MSCs that were previously exposed to BCCs (primed MSCs), or from MSCs that were never exposed to BCCs (naïve MSCs). We overlaid the epigenetic modifiers from these datasets with the following pathways within IPA: neoplasia of cells; proliferation of stem cells; proliferation of cancer cells; and de-differentiation of beta islet cells. The output network identified KMT2B, KMT2D, and DNMT1 as regulators of stem cell genes (Fig. 1A). Since dormant BCCs are functionally similar to CSCs, we proposed that endogenous KMT2B, KMT2D and DNMT1 could be involved in maintaining CSCs [9]. We and others have addressed the role of the cancer niche on dormancy. Based on the information, combined with our other studies showing evidence of cell-autonomous method of dedifferentiation to CSCs, we focused this study to decipher how H3K4 regulate BCCs by cell-autonomous method [50].

### H3K4 methylation in BCC survival

The number of methylation sites on H3K4 could influence cellular functions [53]. We therefore sought the role of H3K4 methylation on BCC quiescence and multipotency with a pan pharmacological inhibitor, WDR5–0103. This inhibitor blunts H3K4 methylation by targeting the core subunit of the KMT2s, WDR5 [54]. Dose-response and time-course studies with BCC viability as readouts identified the optimal conditions as 10 μg/ml of WDR5–0101 and 48 h treatment (Fig. S3). Since WDR5–0103 induced significant (*p < .*05) BCC death, as compared to vehicle (Fig. 1B), we deduced that H3K4 methylation is relevant to BCC survival.

### H3K4 methylation in CSC maintenance

A population of BCCs resisted WDR5–0103 treatment, which led us to conduct studies to gain insights into the how the inhibitor could be affecting the different BCC subsets [8, 9, 55]. BCCs with stable pOct4a-GFP could delineate subsets since GFP intensity is directly proportional to Oct4a levels [9]. Thus, we treated BCCs-pOct4a-GFP with WDR5–0103 for 48 h and then analyzed the surviving BCCs for GFP intensity by flow cytometry, as described [9, 48]. WDR5–0103 significantly (*p* < .05) reduced CSCs (Oct4a^hi^), as compared to vehicle (Fig. 1C). This correlated with an increase of late BC progenitors (Oct4a^neg^) (Fig. 1C), suggesting that the inhibitor induced CSCs to differentiate. Based on this finding, we deduced that loss of cell viability likely occurred in the non-CSC subset, indicating that blunted H3K4 leads to differentiation and cell death.

We next asked if WDR5–0103-mediated decrease of CSCs correlated with reduced levels of multipotent-linked genes. Real time PCR indicated that WDR5–0103 significantly (*p* < .05) decreased Oct4, Sox2 and Nanog mRNA, as compared to vehicle (Fig. 1D). Altogether, pharmacological inhibition of H3K4 methylation indicated its role in preserving the CSC population.

### Synergistic effects of H3K4 inhibitor and carboplatin

Inhibition of H3K4 methylation led to increase percentages of BC progenitors (Fig. 1C), which are mostly cycling cells [9, 18]. This led us to ask if WDR5–0103-mediated differentiation of CSCs would sensitize BC progenitors to carboplatin. We treated BCCs with WDR5–0103 and/or 200 μg/mL carboplatin for 2 days. Carboplatin treatment resulted in ~ 55% cell death as compared to ~ 35% for WDR5–0103 treatment (Fig. 1E). Together, carboplatin and WDR5–0103 showed significant (*p* < .05) increase in cell death as compared to individual treatment (Fig. 1E). Since CSCs have been shown to resist carboplatin treatment, we deduced that increased cell death by carboplatin and WDR5–0103 was partly due to the differentiation effects of WDR5–0103 (Fig. 1F).

### Transcriptomic changes in KMT2B and KMT2D KD BCCs

**Fig. 2:**
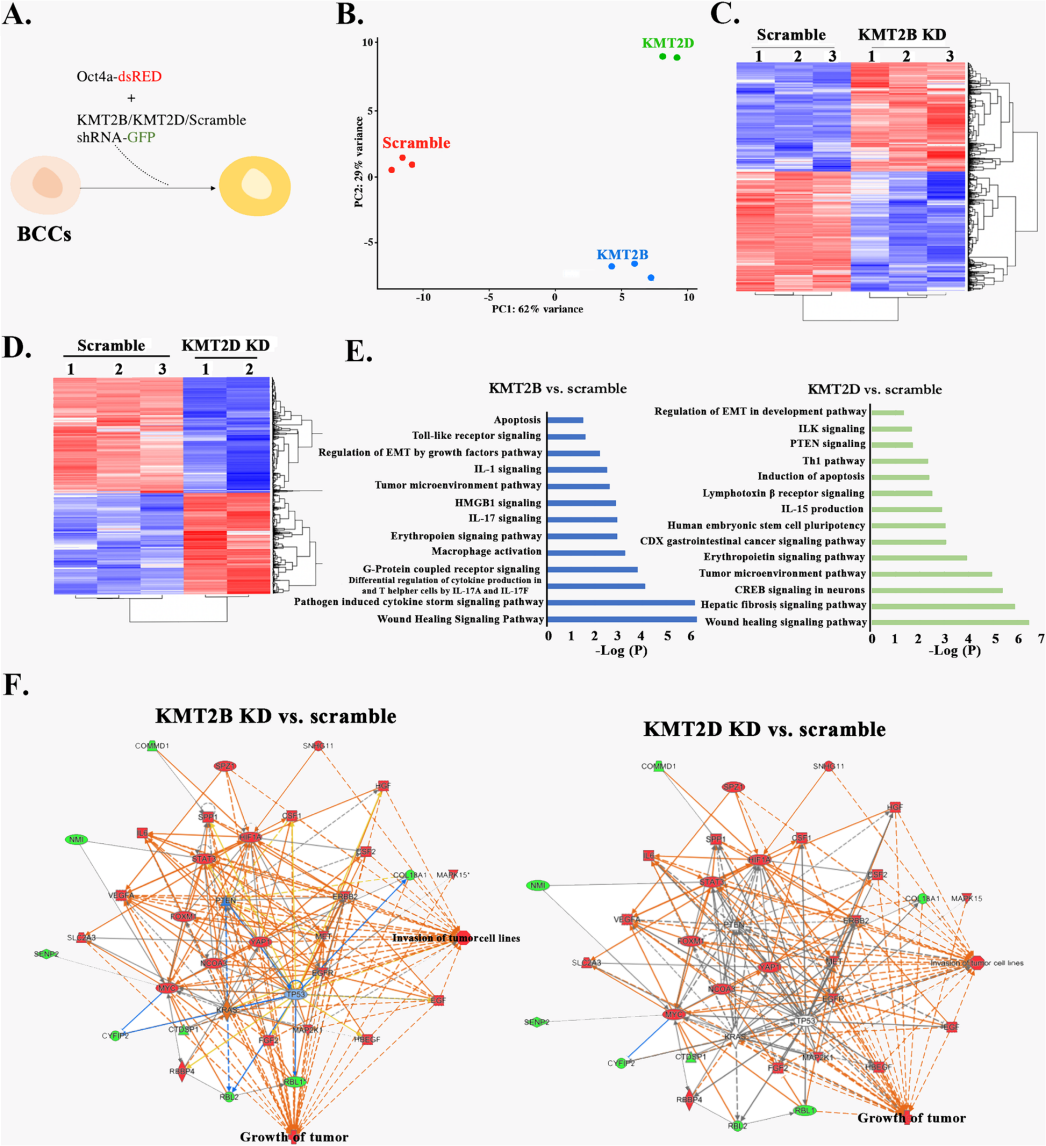
RNA-Seq analyses of KMT2B and KMT2D KD BCCs. **A** Shown is the experimental design in which MDA-MB-231 and T47D expressing Oct4a-dsRed were transduced with KMT2D-GFP tagged shRNA at MOI of 1:50,000. **B** Principal component analysis (PCA) of the different groups of cells that were subjected to RNA-Seq. The plot was established with normalized data (**C** & **D**) Heatmaps of the normalized genes are shown for scramble shRNA and depicting expression differences between scramble and KMT2B (C) or KMT2D KD BCCs. **E** Gene ontology showing canonical pathways upregulated in KMT2B and KMT2D KD BCCs. **F** Genes selected by IPA with functional link to the tumor growth pathway are shown in KMT2B and KNT2D knockdown BCCs. Network show activation of the growth of tumor pathway in botb KD BCCs

The data thus far supported a role for H3K4 methylation in CSC maintenance (Fig. 1). Further, interrogating H3K4 methylation led to CSC differentiation and chemosensitivity (Fig. 1). Since the experimental design contained only BCCs, the findings strongly suggested cell-autonomous method in H3K4 methylation in CSCs. We therefore knocked down KMT2B and KMT2D in BCCs to study the individual role of two H3K4 methylases in CSCs. We performed RNA-seq analyses with the KD BCCs and control with scramble shRNA (Fig. 2A).

PCA of the RNA-seq data showed distinct clustering of the groups with negligible variability of the biological replicates within each group (Fig. 2B). Heatmaps of genes showed distinct expressions between scramble and KMT2B or KMT2D KD BCCs (Fig. 2C and D). Gene ontology (GO) analyses of the RNA-seq data revealed that the upregulated pathways in KMT2B and KMT2D KD BCCs were associated with tumor progression, e.g., inflammatory cues and cell migration (Fig. 2E).

We selected the genes associated with tumor progression and then overlaid them with the dataset linked to tumor growth pathway in IPA. The output revealed that genes associated with tumor growth were enhanced in the KD BCCs, relative to scramble shRNA (Fig. 2F). Collectively, the results showed that KMT2B and KMT2D KD changed the transcriptional landscape of BCCs; particularly, upregulating genes associated with tumor growth. Together, the predicted analyses suggested a loss of a dormant phenotype in the KMT2B and KMT2D KD BCCs.

### Reduced CSCs in KMT2B and KMT2D KD BCCs

**Fig. 3:**
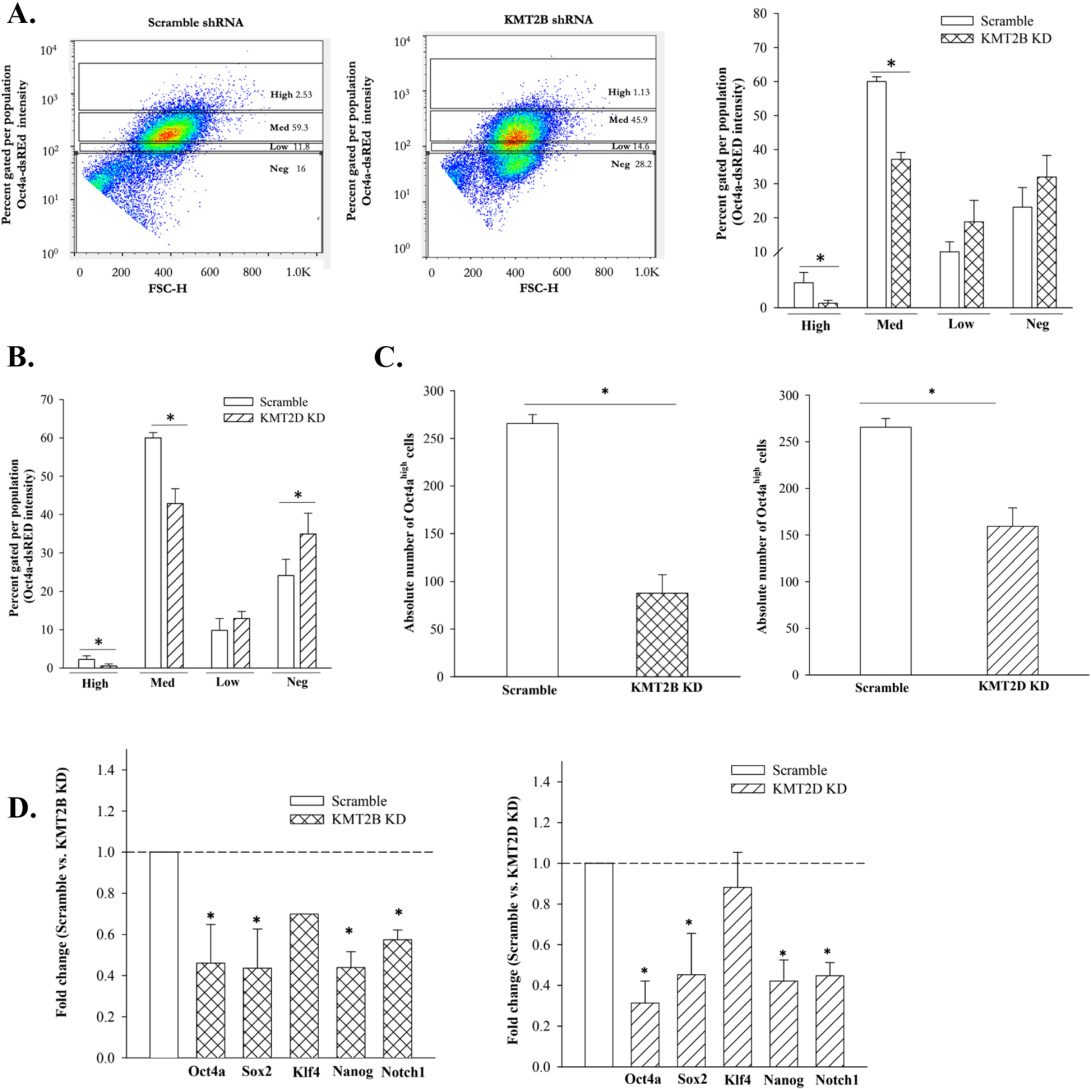
Decreased CSCs in KMT2B and KMT2D KD reduced CSCs but increased BC late-progenitor. **A** MDA-MB-231 BCCs with the Oct4a-dsRED reporter vector and transduced with the KMT2D-GFP shRNA were subjected to flow cytometry to measure the relative dsRED intensity. **B** Percent distribution of BCC subsets after transduction with scramble-shRNA or KMT2D shRNA. **C** Total number of CSCs (Oct4a^high^) in scramble BCCs and KMT2D knockdown BCCs. Each experiment was repeated thrice, where a *p < .05 was considered significant. **D** Reat time PCR for stem cell-associated genes in KMT2B and KMT2D KD BCCs. Control PCR used cDNA from BCCs with scramble shRNA. The value for scramble shRNA is assigned 1 and the experimental values are presented as the fold change. The results show three biological replicates, mean ± SD, *p < .05 vs. scramble shRNA

Analyses of the RNA-seq data showed increases in genes linked to tumor growth in the KMT2B and KMT2D KD BCCs (Fig. 2F). Furthermore, there were increases of BCC progenitors and decreased CSCs after treatment with H3K4 inhibitor (Fig. 1). We therefore asked if this change was specific to KMT2B or KMT2D. We analyzed KD BCCs for subsets by flow cytometry, similar to Fig. 1C [9]. CSCs/Oct4a^hi^ were significantly (*p* < .05) decreased when KMT2B or KMT2D was KD (Fig. 3A and B). The data, when presented as the absolute number of Oct4a^hi^ BCCs showed significant (*p* < .05) decreases, relative to scramble shRNA (Fig. 3C). We also noted a similar decrease for Oct4a^med^ BCCs (Fig. 3A and B). Since Oct4a^hi^ and Oct4a^med^ BCCs were primitive within BCC hierarchy, we deduced that their decrease was due to differentiation [9]. This was corroborated by increases of BCC progenitors (Oct4a^lo^ and Oct4a^neg^) (Fig. 3A and B). Overall, we noted similar results with the pharmacological inhibitor of H3K4 methylase (Fig. 1C).

Next, we asked if decreased CSCs (Fig. 3C) correlated with reduced transcript for stem cell-associated genes. Real time PCR showed significant (*p* < .05) decreases in stem cell transcription factors, Oct4a, Sox2, Klf4, Nanog and Notch 1 in KMT2B and KMT2D KD BCCs, relative to scramble sequence (Fig. 3D). In summary, the findings indicated that KMT2B and KMT2D KD reduced CSCs, and this seemed to be due to differentiation of CSCs to BC progenitors.

### Loss of cycling quiescence in BCCs KD for KMT2B or KMT2D

**Fig. 4:**
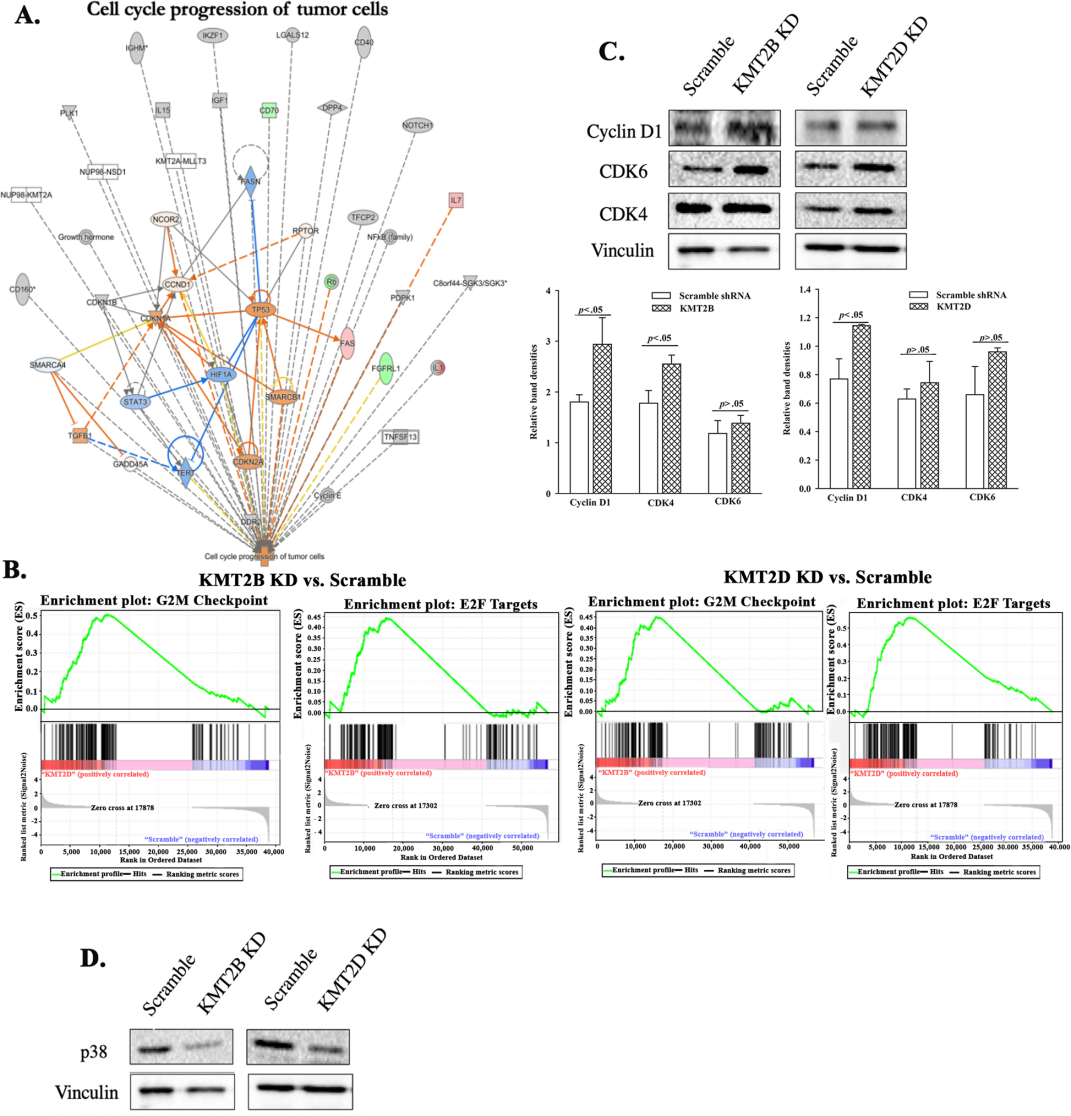
KMT2B and KMT2D in cycling quiescence of BCCs. **A** Overlay of upregulated genes associated in KMT2B and KMT2D knockdown BCCs with cell cycle progression patway in IPA. **B** Gene set enrichment analysis (GSEA) shows upregulation of the E2F targets and G2M checkpoint pathways in KMT2B knockdown and KMT2D knockdown BCCs relative to scramble BCCs. **C** Representative western blot for Cyclin D1, CDK6, and CDK4 in KMT2B or KMT2D KD, and scramble triple positive (T47D) BCCs. Right graphs show the normalized densities for three biological experiments. **D** Western blot for p38 with extracts from KMT2B and KMT2D KD BCCs or BCCs with scramble shRNA

KMT2B and KMT2D KD BCCs have increased progenitors and decreased CSCs (Fig. 3). Induced number of cycling BC progenitors by H3K4 inhibitor was in line with enhanced sensitivity to carboplatin (Fig. 1E and F). We used IPA to determine if KMT2B or KMT2D KD could promote cell cycle progression by overlaying the differentially expressed genes from the RNA-seq data with cell cycle progression pathway. Indeed, the analyses indicated activation of cell cycle progression pathways in KMT2B and KMT2D KD BCCs (Fig. 4A). Gene set enrichment analyses (GSEA) of the RNA-seq data showed a significant enhancement of E2F and G2M cell cycle progression pathways (Fig. 4B). The predicted findings were confirmed in western blot for CDK4, CDK6, and cyclin D1 (Fig. 4C). These increased proteins supported cell cycle transition from G1 to S phase [56].

Reduced CSCs within KMT2B and KMT2D KD BCCs is expected to decrease the ability of BCCs to adapt dormancy [9]. To address this, we examined the KD cells for p38 since its increase has been linked to cellular dormancy [57, 58]. Western blot analyses showed decreases in p38 bands when the extracts were taken from KMT2B and KMT2D KD BCCs, relative to scramble shRNA (Fig. 4D). Altogether, these results indicated that KMT2B or KMT2D KD BCCs led to loss of cycling quiescence.

### Enhanced proliferation and migration by KMT2B and KMT2D KD BCCs

**Fig. 5:**
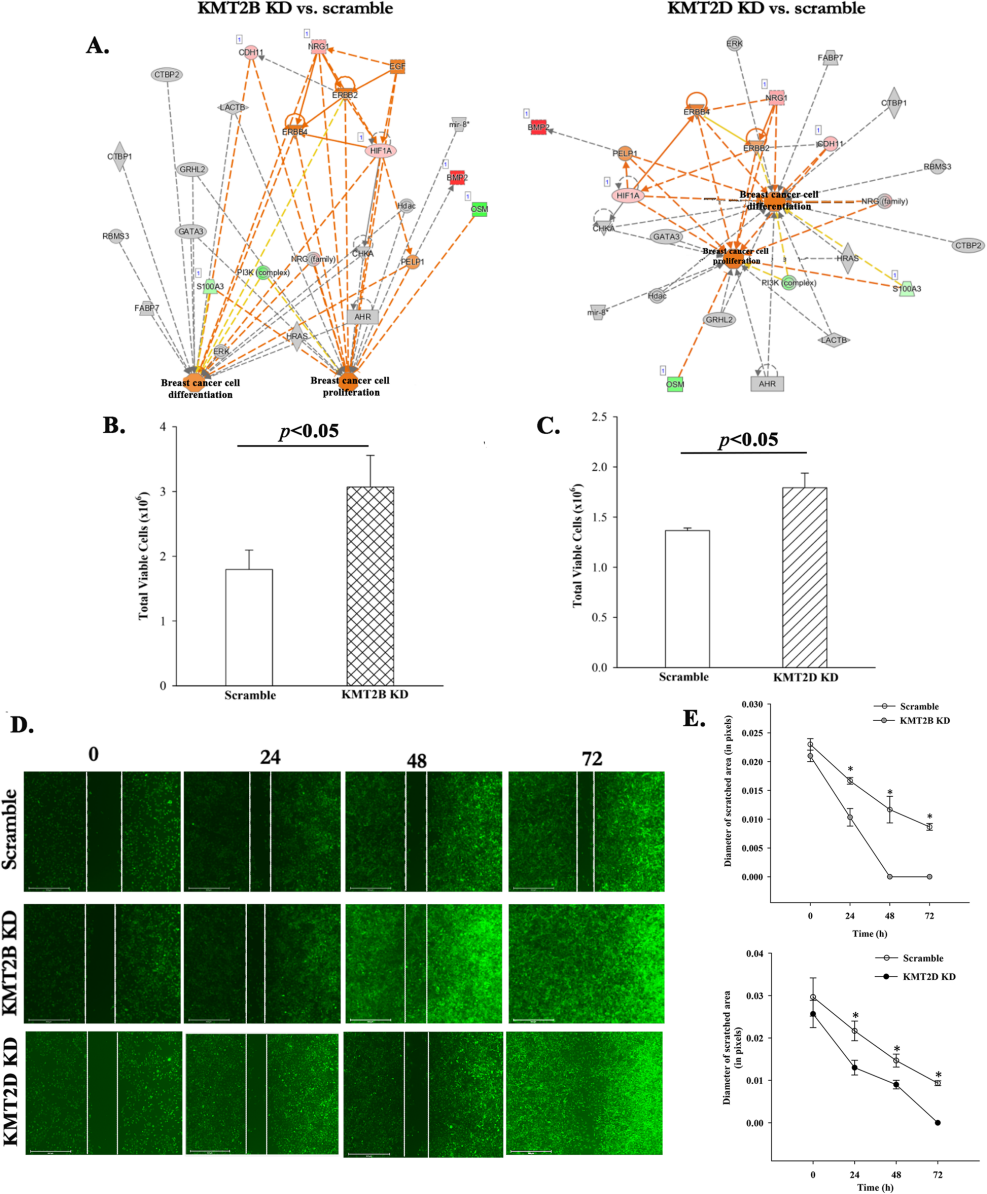
Enhanced proliferation and migration in KMT2 KD BCCs. **A** IPA analyses identified enhanced differentiation and proliferation in KMT2B and KMT2D KD BCCs. **B** & **C** Cell numbers after 48 h for KMT2B (**B**) and KMT2D (**C**) KD BCCs, and BCCs with scramble shRNA sequence. Results are presented as the mean of total viable cells ± SD, *n* = 3, *p < .05. **D** & **E** Shown are representative images of scratch assays with KMT2B and KMT2D KD BCCs (**D**). The scratch areas were quantified and presented as the mean diameter and closure of the scratched area ± SD, *n* = 3, *p < .05 vs the KD BCCs

Increased BC progenitors within the KMT2B and KMT2D KD cells suggested that these two genes could restrict BCC proliferation and favor a dormant state (Figs. 1 and 3). IPA analyses of KD versus scramble shRNA indicated activated pathways linked to proliferation and differentiation (Fig. 5A). We verified proliferation by showing significant (*p* < .05) increases in cell numbers in the KD BCCs, relative to scramble shRNA (Fig. 5B and C).

IPA predicted that KMT2B and KMT2D KD BCCs would show increased cell migration pathways (Figs. S4 and S5). We validated this using scratch assays, and showed increased migration of both KD BCCs, relative to BCCs with scramble shRNA (Fig. 5D and E). While the gap for KMT2B closed at 48 h, similar closure took 72 h for KMT2D BCCs. BCCs with scramble shRNA failed to close the gaps. In summary, the findings demonstrated that KMT2B or KMT2D KD promoted BCC proliferation and migration.

### In vitro *and* in vivo response of KMT2B and KMT2D KD BCCs to chemotherapy

**Fig. 6:**
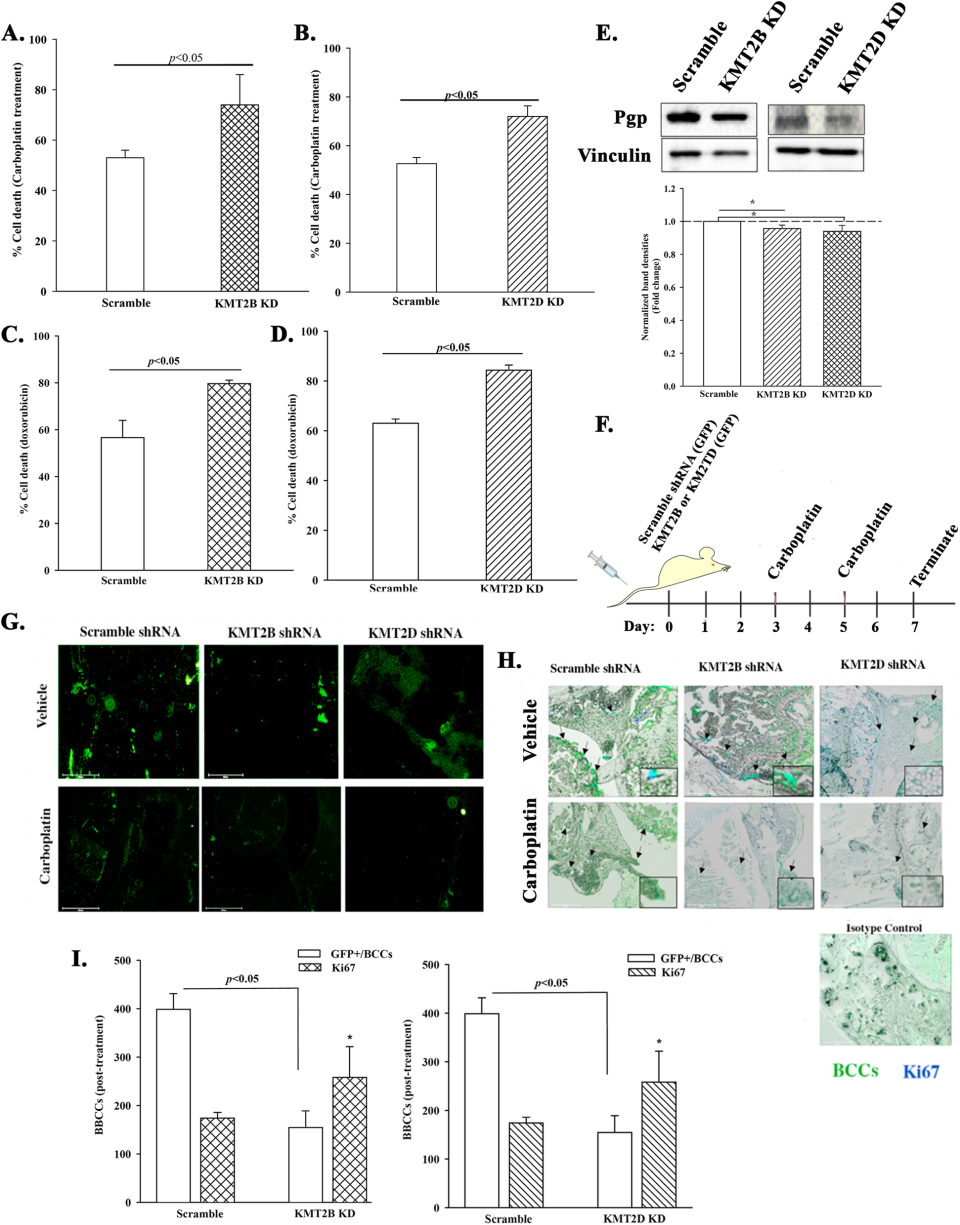
In vitro and in vivo chemosensitivity of KMT2B and KMT2D KD BCCs. **A** & **B** KMT2B (**A**) or KMT2D (**B**) KD BCCs were treated with 200 μg/mL carboplatin for 48 h. Control BCCs were transfected with scramble shRNA and similarly treated. The cells were analyzed with the MTT assay and the results presented as mean ± SD cell death for three biological replicates. Each replicate contained three technical studies. **C** & **D** The studies in `A’ were repeated except for treatment with doxorubicin (1 μM) for 48 h. **E** Western blot for P-gp with extracts from KMT2B and KMT2D KD BCCs, and BCCs with scramble shRNA. The lower graph showed the mean fold change±SD of normalized bands, *n* = 3. * *p* < .05. **F** Diagram showing the in vivo model in which KMT2B or KMT2D KD MDA-MB-231 BCCs were injected in the tail veins of 6-wk female nude mice. Control mice were injected with BCCs containing scramble shRNA. Shown are the timeline (days 3 and 5) treatment with carboplatin (5 mg/kg) or vehicle (1X PBS) on days 3 and 5. The studies were terminated at day 7. **G** Femurs from the euthanized mice in `F′ were scaped at the endosteal region and then immediately examined on an Evos *Fl2* for GPF-positive BCCs. Shown are representative images for five femurs, each from a different mouse. **H** Femurs from the mice described in F and G were decalcified and embedded in paraffin. Sections were analyzed on the Evos *Fl2* for GFP (green) or labeled for Ki67 (blue) (teal cells = green (BCCs) + blue (Ki67). Shown are representative image at 10X magnification. **I** Figures show the total number of GFP+ cells in 10 fields of sections from mouse femurs and KI67+ cells within the GFP+ cells. * *p* < .05 vs. scramble Ki67

We showed mostly progenitors within the KMT2B and KMT2D KD BCCs (Figs. 1, 2, 3, 4 and 5). Since BC progenitors are mostly cycling cells, they are expected to be sensitive to chemotherapy [18]. IPA of the RNA-seq data predicted chemosensitivity of KMT2B and KMT2D KD BCCs versus scramble shRNA (Figs. S6 and S7). Thus, we treated BCCs, KD for KMT2B or KMT2D, or scramble shRNA with carboplatin (200 μg/ml), doxorubicin (1 μM) or vehicle. After 48 h, trypan blue exclusion indicated significant (*p* < .05) cell death in the KMT2B or KMT2D KD BCCs, relative to scramble shRNA (Fig. 6A-D). Enhanced chemosensitivity of KMT2B and KMT2D KD BCCs correlated with decreased of the multidrug-resistant protein Pgp (Fig. 6E).

The in vivo studies used an established model of BC dormancy to test the response of KMT2 KD BCC to carboplatin (Fig. 6F). BCCs, KD for KMT2B or KMT2D, or scramble shRNA (5x10^5^cells in 300 μl PBS) were injected into the tail vein of female nude mice (6 weeks). Our previous studies reported 48–72 h for BCCs to acquire dormancy in BM [9, 19]. We used this time as guide to treat the mice. Mice were inject via intraperitoneal route with vehicle or carboplatin (2 mg/kg) at days 3 and 5. At day 7, mice were euthanized, and the femurs harvested (Fig. 6F). The endosteal region of one femur was scraped to identify BCCs, and the other decalcified for embedding in paraffin. In both analyses, GFP within the shRNA vector served as an indicator of BCCs. The sectioned tissues were labeled for Ki67 as an indicator of cell proliferation.

Fluorescence microscopy of the scraped tissue indicated less BCCs in mice that received the KMT2 KD BCCs and carboplatin treatment (Fig. 6G). Similar treatment of mice with scramble shRNA identified an increase of BCCs (Fig. 6G). Examination of sections from paraffin-embedded femurs indicated higher levels of Ki-67 in KMT2B and KMT2D KD BCCs, relative to scramble shRNA (Fig. 6H and I). Carboplatin treatment reduced Ki67+ BCCs in femurs (Fig. 6G-I). The results also showed continued proliferation, based on Ki67 even after carboplatin treatment. Overall, the in vivo and in vitro studies corroborated chemosensitivity of KMT2 KD BCCs.

### Chemosensitivity of KMT2B and KMT2D KD BCCs in brain

**Fig. 7:**
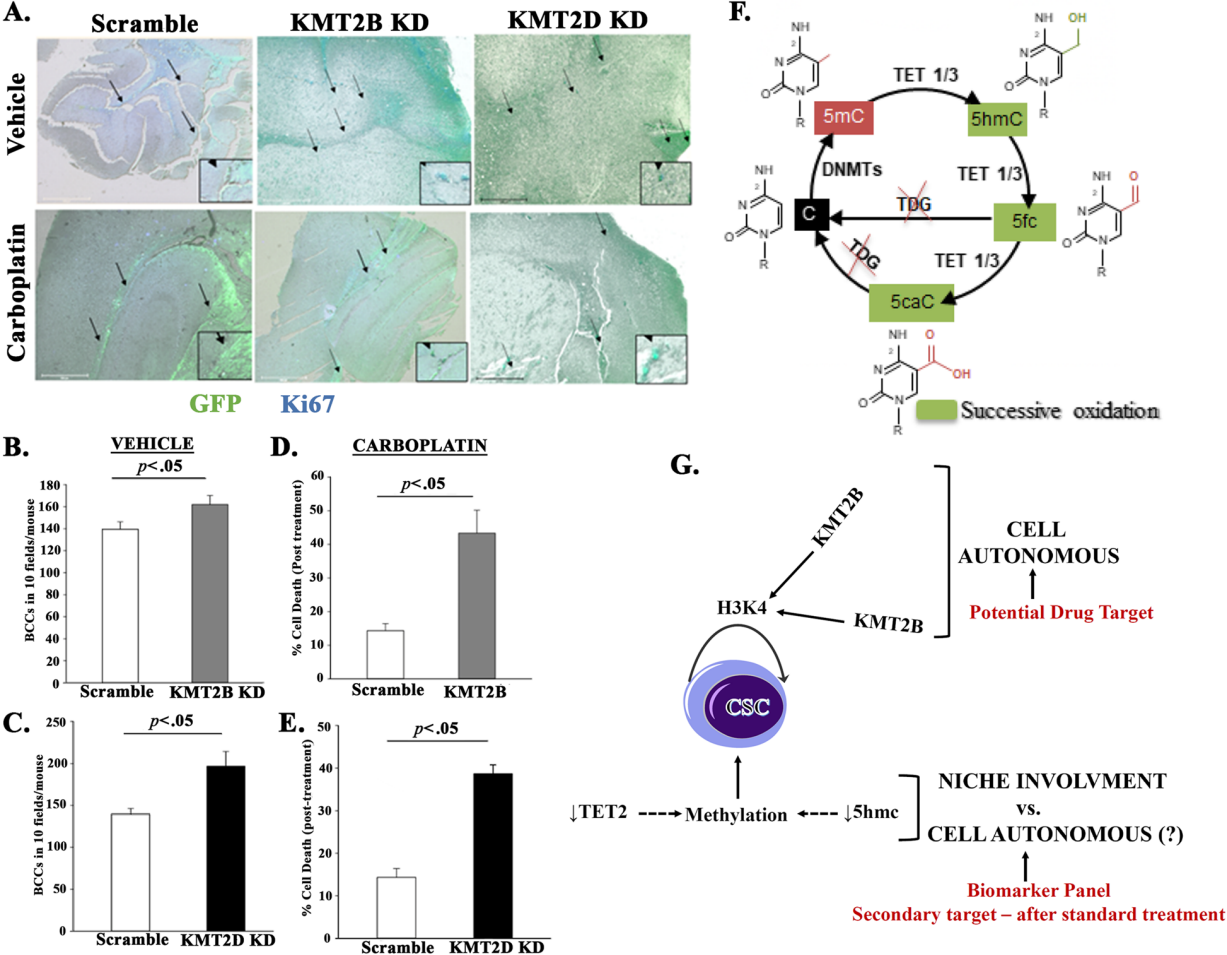
KMT2B and KMT2D KD promote tertiary metastasis to the brain. **A** MDA-MB-231 BCCs (5 × 10^5^) with scramble sequence-GFP, KMT2B KD-GFP, and KMT2D KD-GFP were injected intravenously into nude female mice as for Fig. 6. On day 7, the brain was harvested, and the paraffin-embedded slides were imaged to assess for BCCs (green) and for Ki67 cells (teal cells: green/BCCs + blue/Ki67). Image magnification: 100x. **B** and **C** The total number of BCCs for three mice treated with vehicle were counted in 10 fields per section. The data are presented as the mean ± SD total number of viable cells in brains of mice injected with KMT2B (**B**) or KMT2D (**C**) KD, or scramble shRNA. **D** and **E** Percent cell death after carboplatin treatment was calculated with the total number of BCCs for vehicle set as 100% viablity. The data for KMT2B (**D**) and KMT2D (**E**) are calculated as the mean ± SD. **F** DNA methylation and oxidation cycle: Genomic DNA is methylated (5mC) by DNA methyltransferase enzymes (DNMTs). TET enzymes (TET1, TET2, TET3) can successively oxidize 5mC into 5hmC, 5fC and 5caC. Subsequently, 5fC and 5caC can be excised by TDG and further repair by the Base Excision Repair (BER) system leading to DNA de-methylation. **G** Shown is a overall summary of the findings. The cartoon indicates a cell-autonmous role for H3K4 with the data supporting potential drug target. The outcome on DNA methylation has not proved to be cell autonomous. The data however suggested the inclusion in a biomarker panel and follow-up treatment

We previously reported on brain metastasis when dormant MDA-MB- 231 BCCs were induced to reverse dormancy [48]. We also showed that BCCs that exited dormancy were chemosensitive, resulting in reduced brain metastasis [48]. Since the experimental evidence indicates that KMT2B and KMT2D maintain CSCs, we asked if their KD could recapitulate reverse dormancy (transition out of cellular quiescence), and if treated, this should lead to significant mitiagation of brain metastasis. We evaluated the brain sections of the mice that were treated with vehicle or carboplatin (Fig. 6F). We counted 10 fields per section in three mice and then calculated the total number of BCCs in the brain. This resulted in significantly (*p* < .05) more KMT2B and KMT2D KD BCCs in the vehicle treated mice, relative scramble shRNA (Fig. 7B and C). We counted the total number of BCCs in the carboplatin treated mice and used the values obtained in the brain of vehicle treated as 100% to calculate cell death in brain. The results indicated significant (*p* < .05) cell death with the knockdown cells, relative to scramble (Fig. 7D and E). In summary, the results showed that targeting H3K4 could reduce brain metastasis.

### Epigenomic maintenance of CSC in BC patients

Along with KMT2B and KMT2D, DNMT is predicted to be involved in multipotency of CSCs (Fig. 1). To test the involvement of DNMT in CSC maintenance, we focused on the associated regulatory genes (Fig. 7F). Specifically, on TET2 since it can oxidize 5mC, leading to demethylation [59] (Fig. 7F). Additionally, we previously reported on increased TET2 during the first phase of dedifferentiation of BCCs that were exposed to microenvironmental exosomes [18]. TET 2 is reduced during the second phase as differentiation complete, suggesting that retained methylation is relevant to sustained stemness in BCCs. The question is how DNA methylation, along with what the above findings on methylation of H3K4, could be combined to sustain BCC stemness. We tested pan-cytokeratin+ cells in the blood of BC patients for genes associated with stem cell and DNMT (Tables 1 and 2).

**Table 2:** Phenotype of circulating cytokeratin + cells

Patients	Cytokeratin %	EpCam %	Oct4a %	TET2%	5hmC %	CSC Support	Treatment
1	.85	.085	.09	Not Done	Not Done	Yes	Yes
2	5.92	.03	.02	.2	4.81	No	No
3	.24	.04	.04	0	.23	Possible	Yes
4	.79	.62	.76	0	0	Yes	Yes
5	9	.51	.67	.39	0	Yes	Yes
6	15.1	.39	6.3	.1	0	Yes	Yes
7	.46	.01	.26	0	0	Yes	Yes
8	.89	.79	.27	0	.85	No	Yes

Cytokeratin + cells were analyzed by gating the nucleated fraction in peripheral blood. The identified cytokeratin+ cells were further analyzed for the shown markers. CSC support was determined by analyses of the combined phenotype - EpCam and Oct4a as potential stemness, and further sustenance of CSCs with decreases in TET2 and/or 5hmC, which prevent demethylation

As noted in the patient demographics, most subjects were treated anti-cancer regimen that caused low blood counts; hence the limited amount of blood (Table 1). We however obtained sufficient blood samples to analyze two panels of combined antibodies. We gated the pan cytokeratin + cells then used Panel 1 antibodies to analyze EpCAM and Oct3/4, and Panel 2 to assess Oct3/4, TET2 and 5hmC (hydroxylation) (Fig. 7F).

Patient 2 blood, which was taken before treatment, showed high levels of 5hmC, detectable TET2, and low levels of stem cell-associated Oct3/4 and EpCAM (Table 2) [8]. Together, this profile suggested that the patient could be in the initial phase of dedifferentiating to CSCs but does not show support fir CSC maintenance. Since this blood was tested before treatment, the data confirmed expected heterogeneity of circulating BCCs. Specifically, prior to treatment, it is expected that there will be excess BC progenitors, relative to CSCs. We propose that the BC in this patient could be at the initial phase of dedifferentiation because TET2 has been shown to be needed for step 1 dedifferentiation [18].

Insufficient blood sample for Patient 1 allowed us to limit our analysis with Panel 1 antibodies. The comparable percentages of Oct4 and EpCAM suggested the presence of circulating CSCs. Patient 6 who was 89 years at analyses, was on an estrogen modulator. The blood of this patient showed the highest percentage of cytokeratin + cells that were positive for EpCAM and Oct3/4, and undetectable 5hmC, which strongly suggested methylation of increased CSCs. We deduced that Patient 8 showed limited support for CSCs, despite undetectable TET2 due to increased 5hmC, which could be linked to other TET proteins (Fig. 7F). In summary, this section indicated that in addition to H3K4, 5mC and TET 2 could be indicators to assess sustained CSCs in BC patients during treatment.

Finally, we used the last two blood samples (Patients 9 and 10) to determine if it could be possible to induced CSC differentiation in patients (Table 3). If so, this could indicate a potential intervention following standard treatment, which would prolong cancer recurrence. We treated the mononuclear cells with carboplatin and/or azacitidine for 7 days. Analyses of the mononuclear cells with Panels 1 and 2 antibodies indicated that Patient 9, who was treated, showed evidence of CSC-like in the blood. However, azacitidine led to detectable DNA hydroxylase to methylate the DNA, which is consistent with BC progenitors. Patient 10, who was presented with triple negative BC showed mixed population of CSCs and demethylated BCCs, regardless of treatment. Overall, these results with patient samples are intriguing and requires a more expanded investigation,

**Table 3:** Timeline changes in cytokeratin + cells in patients’ PBMCs

In vitro Treatment	Cytokeratin %	EpCam %	Oct4%	Tet2%	5hmC %	Remarks
Patient 9						MNCs with vehicle showed reduced CSCs. Double treatment led to enhanced DNA methylation
Vehicle	.06	.02	.02	0	0	
Carboplatin	.34	.29	.01	.06	.06	
Carboplatin + Azacitidine	.33	.13	.04	.07	.19	
Patient 10						Treatment of triple negative BCCs reduced DNA hydroxylase
Vehicle	.48	.04	.03	.03	.39	
Carboplatin	.2	.04	.06	.02	.06	
Carboplatin + Azacitidine	.45	.15	.04	.05	.06	

Table showed the results of Patients 9 and 10, untreated and treated with the shown drugs. Mononuclear cells (MNCs) were treated with carboplatin and/or azacitidine. At day 7, the cells were analyzed by phenotype with the two panel antibodies described in Table 2

## Discussion

This study reports on a cell-autonomous method elicited by H3K4 methylation to sustain multipotency in CSCs (Figs. 1, 2, 3, 4, 5 and 6). This role of H3K4 methylation appears to be aided by DNMT (Table 1, Fig. 1A). In a previous study in which we examined the role of MSC-derived exosomes on BC dedifferentiation, we identified DNMT as a regulator of CSC maintenance (Fig. 1A) [18]. More importantly, we noted an indirect relationship between markers of stemness and DNMT associated proteins in cytokeratin + cells from treated BC patients (Table 1). The relevance of this study indicated that DNA methylation rather than its hydroxylation is important in CSCs. This was an intriguing observation since evidence of DNA hydroxylation has been shown to be relevant during the initial phase of BCCs dedifferentiating to CSCs [18]. This is line with a need for gene transcription during the early phase as BCCs begin to dedifferentiate and once the cells attain multipotency/CSCs, transcription is diminished to attain quiescence.

Since dormant BCCs are similar to CSCs [9], the present findings provide insights into a different method by which BCCs attain dormancy. In this case, we showed a mechanism of cell autonomy through H3K4 methylases. Similar BCC quiescence can be supported in the BM by microenvironmental cells such as MSCs, macrophages and stromal fibroblasts [19, 26]. Indeed, H3K4 methylation marks have been proposed as drug targets [60]. However, the question is how epigenetic targeted drugs should be used to treat BC. Of course, this study showed a role for H3K4 and perhaps DNA methylation in CSC maintenance. We do not propose to change the standard of care, but to monitor circulating BCCs as a functional indicator for targeted treatment to prolong BC remission. Studies with hematological cancer showed that induced differentiation of leukemia stem cells with bortezomib could induce chemosensitivity [61]. Despite the limited number of analyzed BCCs in blood (Table 1), the evidence showed intriguing information that similar analyses could be used in expanded studies to guide how BC patients are treated.

We were intrigued by the results of the small studies with patient blood (Table 1). The data showed circulating CSC-like cytokeratin+ BCCs in the otherwise aggressive Her2+ BC (Table 1). The data indicated that CSCs could be sustained with concomitant decrease of TET2. The latter decrease indicated reduced DNA hydroxylation to maintain DNA methylation. This small study was conducted with patients from the University Hospital with economically disadvantage population. It is not unusual for these patients to be first diagnosed with late-stage BC. Due to limited samples and number of patients who consented, we took the opportunity to use Patients 9 and 10 samples to examine if cytokeratin + BCCs from patients who were undergoing standard treatment could be induced to chemosensitivity. As expected, based on the outcome shown in Table 3, Patient 9 showed CSC-like with vehicle treatment, which changed when the mononuclear cells were challenged with azacitidine. This led to increases in TET2 and 5hmc indicating demethylation, which would increase differentiation (Fig. 1). Patient 10 who was diagnosed with triple negative BC started with heterogeneous BCCs, which remained similar with azacitidine (Table 3). The latter findings indicated that similar studies are required with more indepth studies and experimental designs since this type of analyses could be used as biomarkers of treatment. The data discussed in the previous paragraph for leukemia, combined with this study indicated that this should require a large study with global impact. This will help address healthcare disparities in BC worldwide. Our findings shed therapeutic insight across global markets marked by healthcare inequities. In the meantime, drugs are available that could be repurposed to target the CSCs after standard treatment. This is important for long-term remission rather than short recurrences into metastatic BC.

Targeting KMT2s shared WDR5 core subunit with WDR5–0103, led to cell death, as well as differentiation with chemosensitivity (Fig. 1) [54, 62]. Studies with the pharmacological agent as well as the knockdown studies provided insights into how CSCs are maintained in dormancy. The experimental studies indicated that H3K4 methylation is important to maintain CSCs while preventing differentiation to chemosensitive BCCs (Fig. 1). The increased BC progenitors after pharmacological targeting of H3K4 was due to both KMT2B and KMT2D, based on the KD studies.

The in vitro findings, were tested in vivo in nude mice with an established model of BC dormancy in the BM (Fig. 6) [9]. We noted sensitivity to carboplatin when KMT2B and KMT2D KD BCCs were injected in mice (Fig. 6). This was interesting because these BCCs were in an environment of MSCs that can release exosomes with H3K4 methylase. Thus, the findings indicated that KMT2B and KMT2D could be relevant drug targets. Future studies are needed to determine how the treatment could be done safely since the BM is home to hematopoietic stem cells that are likely to share similar H3K4 marks. Similarly, if the findings in Tables 1 and 3 could be guides to treatment, this would require safety studies.

Most chemotherapies target rapidly proliferating cells more efficiently than quiescent dormant BCCs [63]. We noted synergism between carboplatin and WDR5–0103 with respect to cell death (Fig. 1E). This was partly explained by WDR5–0103 being able to differentiate CSCs into proliferating BC progenitors (Fig. 1). WDR5–0103 inhibits global H3K4 methylation by targeting all members of the KMT2 family [54]. However, among the KMT2 family, data using previous exosomal cargo that dedifferentiated BCCs to CSCs, predicted roles for KMT2B and KMT2D (Fig. 1A). Using knockdown studies, the data supported roles for KMT2B and KMT2D in sustained stemness in CSCs.

Insights into a strong role for cell-autonomous regulation of CSCs were derived from bioprinting of BCCs in methylcellulose ink [50]. The printing occurred without endogenous BM cells, which led us to propose that the BCCs could survive with epigenomic changes. This bioprinting study led us to reanalyze the exosomal cargo from MSCs (Fig. 1A) [18]. RNA-seq data from KMT2B and KMT2D KD BCCs further supported cell-autonomous regulation. We noted upregulation of canonical pathways linked to cancer growth and proliferation when these two H3K4 methylases were knocked down (Figs. 2 and 4). These findings, together with the functional studies, indicated that cell-autonomous support of CSC by KMT2B and KMT2D sustain dormancy and drug resistance [9].

KMT2B KD upregulated inflammatory pathways such as interleukin (IL)-1 signaling, Toll-like receptor signaling, and pathogen-induced cytokine storm in BCCs (Fig. 2). KMT2D KD in BCCs resulted in activation of IL-signaling pathway and T-helper 1 signaling pathway (Fig. 2). There is a strong association between the upregulation of pro-inflammatory pathways and dormancy reversal [64, 65]. Disruption of direct cellular communication between BCCs and BM MSCs enhanced IL-1 secretion from the latter cells to mediate BCC proliferation [66]. Furthermore, the IL-1 signaling pathway promotes cancer cell proliferation and angiogenesis through the activation of the NFκB [67]. Importantly, toll-like receptor signaling and pathogen-induced cytokine storm are two factors that can induce dormancy reversal [49, 68–70]. Toll-like receptors recognize pathogen-associated and endogenous damage-associated molecular patterns to trigger a pro-inflammatory immune response that result in pathogen clearance [71]. Collectively, the RNA-seq findings indicated that pathways associated with inflammation are enhanced in KMT2B and KMT2D KD BCCs.

GSEA of the RNA-seq dataset confirmed the functional studies by showing that E2F targets and G2M checkpoint pathways are enriched in KMT2B and KMT2D KD BCCs, as compared to scramble BCCs (Fig. 4). The E2F transcription factors promote cell cycle progression by inducing cell entry into S-phase [72–74]. The G2/M checkpoint prevents mitosis initiation if the cells present DNA damage [75]. The transition of cells from the G2-phase to the M-phase of the cell cycle is consistent with cell proliferation [76]. Activation of cell cycle progression in KMT2 KD BCCs was accompanied by increased proliferation and migration, which is in line with reverse dormancy (Fig. 4). There was a 24 h difference between KMT2D and KMT2B KD BCCs with respect to gap closure in the scratch assay (Fig. 5D and E). This could be attributed to the fact that these two proteins, although members of the KMT2 family, perform different H3K4 methylation modifications and this might contribute to gene expression variability [26].

KMT2B and KMT2D KD enhanced BCC metastasis to the brain, which is consistent with dormancy reversal [48]. However, carboplatin treatment significantly reduced KMT2B and KMT2D KD BCCs in the brain (Fig. 7). Previous studies have indicated that carboplatin can be used to treat tumors that have compromised the integrity of the blood-brain barrier [77, 78]. Thus, it is possible that carboplatin was able to eliminate the KMT2 KD BCCs in the brain since these cells might have compromised the blood-brain barrier.

Although the main in vivo studies conducted established BC dormancy to the BM through intravenous injection, the model validated BC metastasis to the BM. This was deduced in studies in which we injected BCCs into the mammary fat pad of mice and then examined the femur before the tumor grew to recapitulate early dormancy (Fig. S8). The early detection of high GFP+ BCCs indicated early migration to femurs. In summary, this study reported on a cell-autonomous method to maintain CSCs but showed that reversed methylation could chemosensitize the otherwise resistant CSCs. The study also showed a role for DNMT associated proteins as potential markers to guide treatment of BC patients following standard care. We show that circulating BCCs can be a functional indicator for targeted treatment to improve the durability of remission in BC. These findings are summarized in Fig. 7G, which shows proved cell-autonomous role for H3K4 to maintain CSCs. Although the patients’ data, and computational studies (Fig. 1A) suggest similar role for DNMT, it is unclear if there is an involvement of the tissue niche. The data also supported that drugs targeting H3K4 methylation could be potential for future treatment. The outcome on DNA methylation suggested that the test markers with future inclusion of epigenetic genes could be part of a biomarker panel to predict treatment outcome.

### Supplementary Information

**Additional file 1: Fig. S1.** Generation of BCCs, knockdown for KMT2B. (A) Experimental design: BCCs were stably transfected with pOct4a-dsRed and transduced with the KMT2B-GFP tagged shRNA at a multiplicity of infection of 1:50,000. The image was created with BioRender. (B) Western blot shows knockdown efficiency in clones A and C. (C) Representative images demonstrate transduction efficiency with scramble and KMT2B shRNA compared to wild-type MDA-MB-231. **Fig. S2.** Generation of BCCs, knockdown for KMT2D (A) Experimental design: MDA-MB-231 expressing Oct4a-dsRed and T47D (not shown) were transduced with KMT2D-GFP tagged shRNA with an MOI of 1:50,000. The image was created with BioRender. (B) Representative images show transduction efficiency with scramble and KMT2B shRNA compared to untransfected MDA-MB-231. **Fig. S3.** Inhibition of H3K4 methylation decreases BCC viability. (A) Experimental design: BCCs were exposed to WDR5–0103 for 48 h. (B) MDA-MB-231 BCCs were exposed to multiple doses (5 μg/ml, 10 μg/ml, and 20 μg/ml) of WDR5–0103 for 48 h, and viability was assessed with Trypan blue exclusion. (C) Viable MDA-MB-231 BCCs were counted after exposure to WDR5–0103 (10 μg/ml) by Trypan blue staining. Each experiment was repeated thrice, where *p < 0.05 was considered significant. **Fig. S4**. KMT2B knockdown activates genes linked to the migration of BCC lines. IPA network shows upregulation of genes related to the migration of BCCs upon KMT2B knockdown. **Fig. S5.** KMT2D knockdown promotes the activation of genes involved in BCC migration. IPA analysis using the RNA-seq data shows that KMT2D knockdown upregulates genes implicated in BCC migration. **Fig. S6.** KMT2B knockdown reduces chemotherapy resistance in BCCs. In silico analyses illustrate that KMT2B knockdown inhibits chemotherapy resistance in BCCs. **Fig. S7.** KMT2D knockdown downregulates genes involved in chemotherapy resistance. IPA network shows that KMT2D knockdown reduces chemotherapy resistance in BCCs. **Fig. S8.** Orthotopic route of BC dormancy in bone marrow. MDA-MB-231 BCCs with pOct4a-GFP (5 × 10^5^) were injected into the mammary fat pad of female (6 weeks) nude BALB/c. After 1 week, the mice were euthanized, and the femurs were harvested and scraped to evaluate the presence of BCCs. Shown is the fluorescence microscopy for GFP cells in the femurs of two mice. Representative image of a femur that was not injected with BCCs is shown at left. The slides were imaged with the EVOS FL AUTO 2 microscope at a magnification of 200X.

## Data Availability

No datasets were generated or analysed during the current study.
