# Supplementary material for: Revealing role of epigenetic modifiers and DNA oxidation in cell-autonomous regulation of Cancer stem cells
Source: Cell Commun Signal. 2024 Feb 12;22:119. doi: 10.1186/s12964-024-01512-1 (PMC10863086; doi:10.1186/s12964-024-01512-1)
Supplement: Supplementary file 1 — Additional file 1: Fig. S1. Generation of BCCs, knockdown for KMT2B. (A) Experimental design: BCCs were stably transfected with pOct4a-dsRed and transduced with the KMT2B-GFP tagged shRNA at a multiplicity of infection of 1:50,000. The image was created with BioRender. (B) Western blot shows knockdown efficiency in clones A and C. (C) Representative images demonstrate transduction efficiency with scramble and KMT2B shRNA compared to wild-type MDA-MB-231. Fig. S2. Generation of BCCs, knockdown for KMT2D (A) Experimental design: MDA-MB-231 expressing Oct4a-dsRed and T47D (not shown) were transduced with KMT2D-GFP tagged shRNA with an MOI of 1:50,000. The image was created with BioRender. (B) Representative images show transduction efficiency with scramble and KMT2B shRNA compared to untransfected MDA-MB-231. Fig. S3. Inhibition of H3K4 methylation decreases BCC viability. (A) Experimental design: BCCs were exposed to WDR5–0103 for 48 h. (B) MDA-MB-231 BCCs were exposed to multiple doses (5 μg/ml, 10 μg/ml, and 20 μg/ml) of WDR5–0103 for 48 h, and viability was assessed with Trypan blue exclusion. (C) Viable MDA-MB-231 BCCs were counted after exposure to WDR5–0103 (10 μg/ml) by Trypan blue staining. Each experiment was repeated thrice, where *p < 0.05 was considered significant. Fig. S4. KMT2B knockdown activates genes linked to the migration of BCC lines. IPA network shows upregulation of genes related to the migration of BCCs upon KMT2B knockdown. Fig. S5. KMT2D knockdown promotes the activation of genes involved in BCC migration. IPA analysis using the RNA-seq data shows that KMT2D knockdown upregulates genes implicated in BCC migration. Fig. S6. KMT2B knockdown reduces chemotherapy resistance in BCCs. In silico analyses illustrate that KMT2B knockdown inhibits chemotherapy resistance in BCCs. Fig. S7. KMT2D knockdown downregulates genes involved in chemotherapy resistance. IPA network shows that KMT2D knockdown reduces chemotherapy resistance in BCCs. Fig. S8. Orth [file 12964_2024_1512_MOESM1_ESM.docx]

**Supplemental Information**


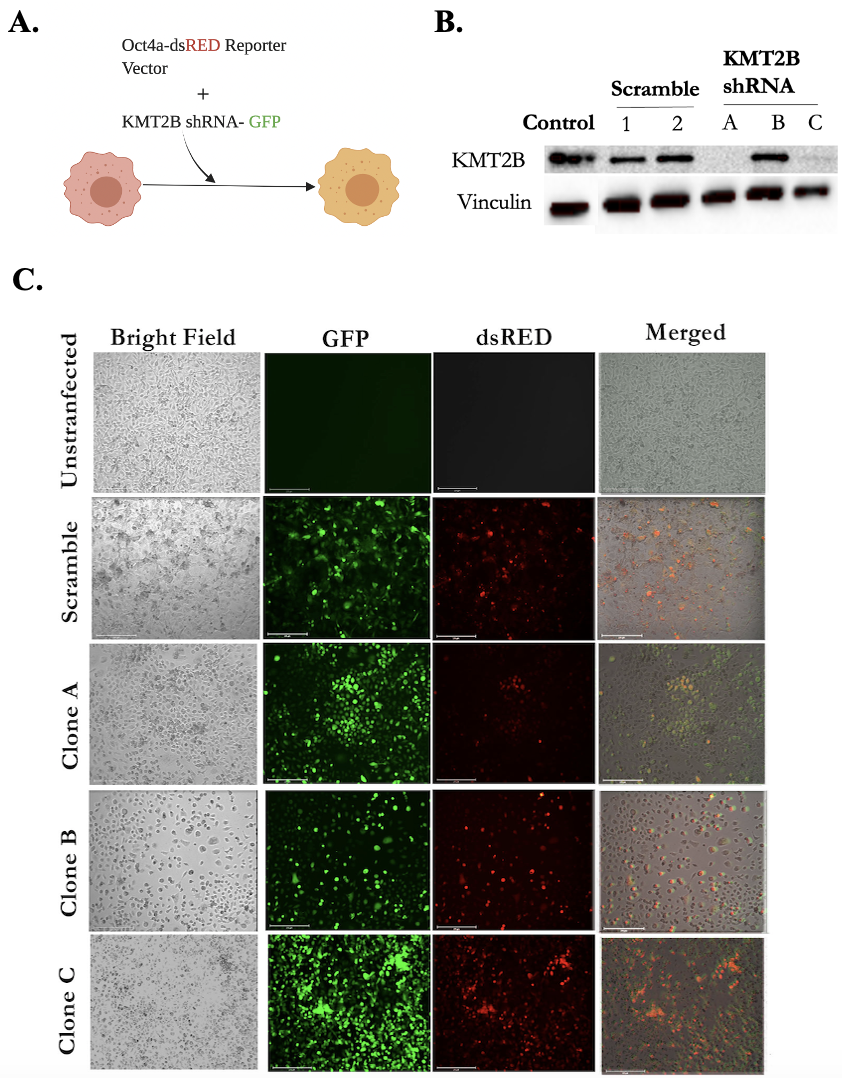


**Figure S1. Generation of BCCs, knockdown for KMT2B. (A)** Experimental design: BCCs were stably transfected with pOct4a-dsRed and transduced with the KMT2B-GFP tagged shRNA at a multiplicity of infection of 1:50,000. The image was created with BioRender. **(B)** Western blot shows knockdown efficiency in clones A and C. **(C)** Representative images demonstrate transduction efficiency with scramble and KMT2B shRNA compared to wild-type MDA-MB-231.


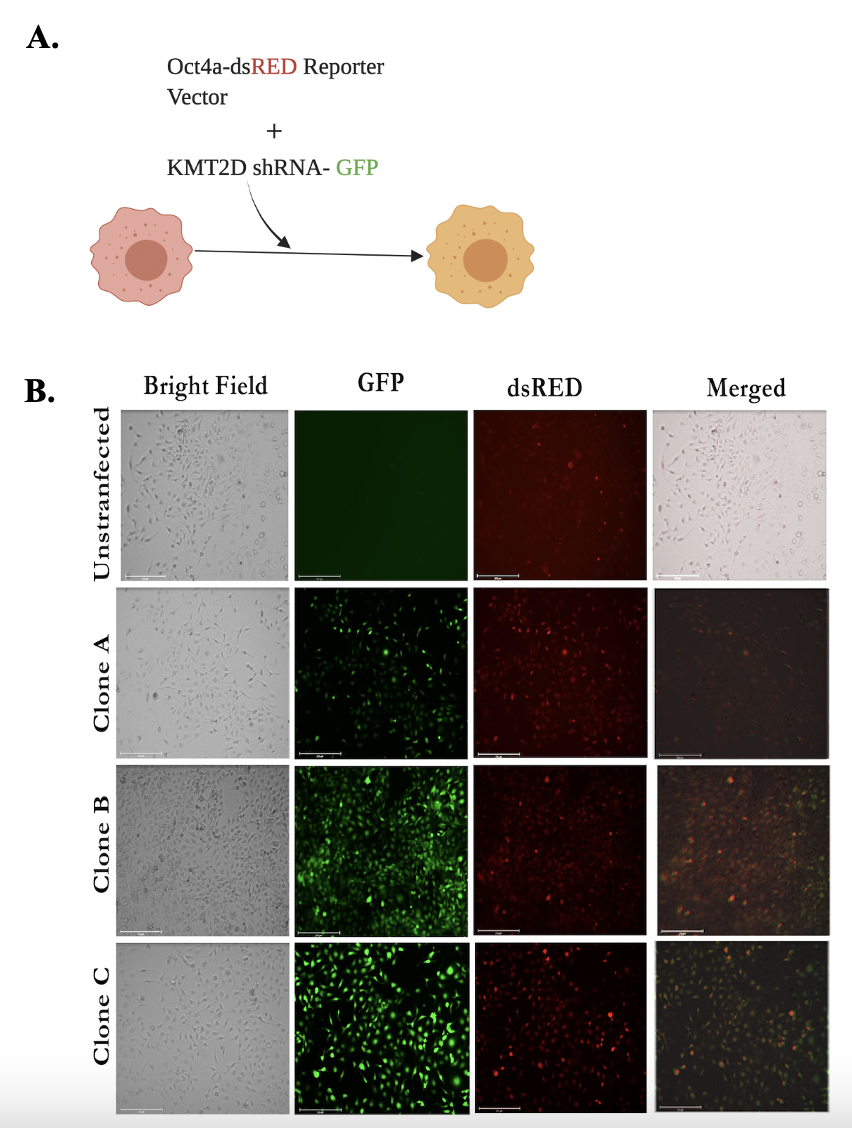


**Figure S2. Generation of BCCs, knockdown for KMT2D (A)** Experimental design: MDA-MB-231 expressing Oct4a-dsRed and T47D (not shown) were transduced with KMT2D-GFP tagged shRNA with an MOI of 1:50,000. The image was created with BioRender. **(B)** Representative images show transduction efficiency with scramble and KMT2B shRNA compared to untransfected MDA-MB-231.


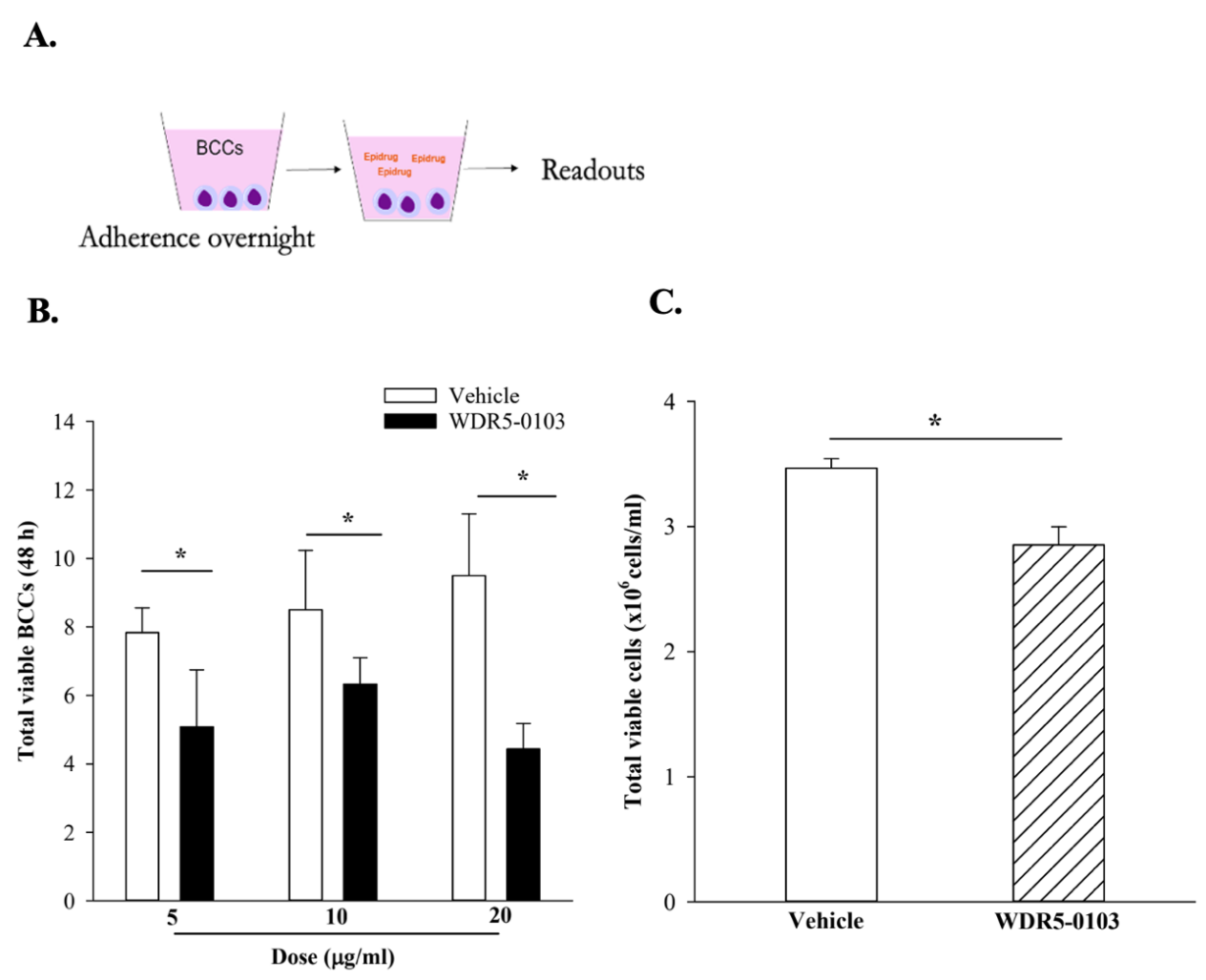


**Figure S3. Inhibition of H3K4 methylation decreases BCC viability. (A)** Experimental design: BCCs were exposed to WDR5-0103 for 48 h. **(B)** MDA-MB-231 BCCs were exposed to multiple doses (5 μg/ml, 10 μg/ml, and 20 μg/ml) of WDR5-0103 for 48 h, and viability was assessed with Trypan blue exclusion. **(C)** Viable MDA-MB-231 BCCs were counted after exposure to WDR5-0103 (10 μg/ml) by Trypan blue staining. Each experiment was repeated thrice, where **p*<0.05 was considered significant.


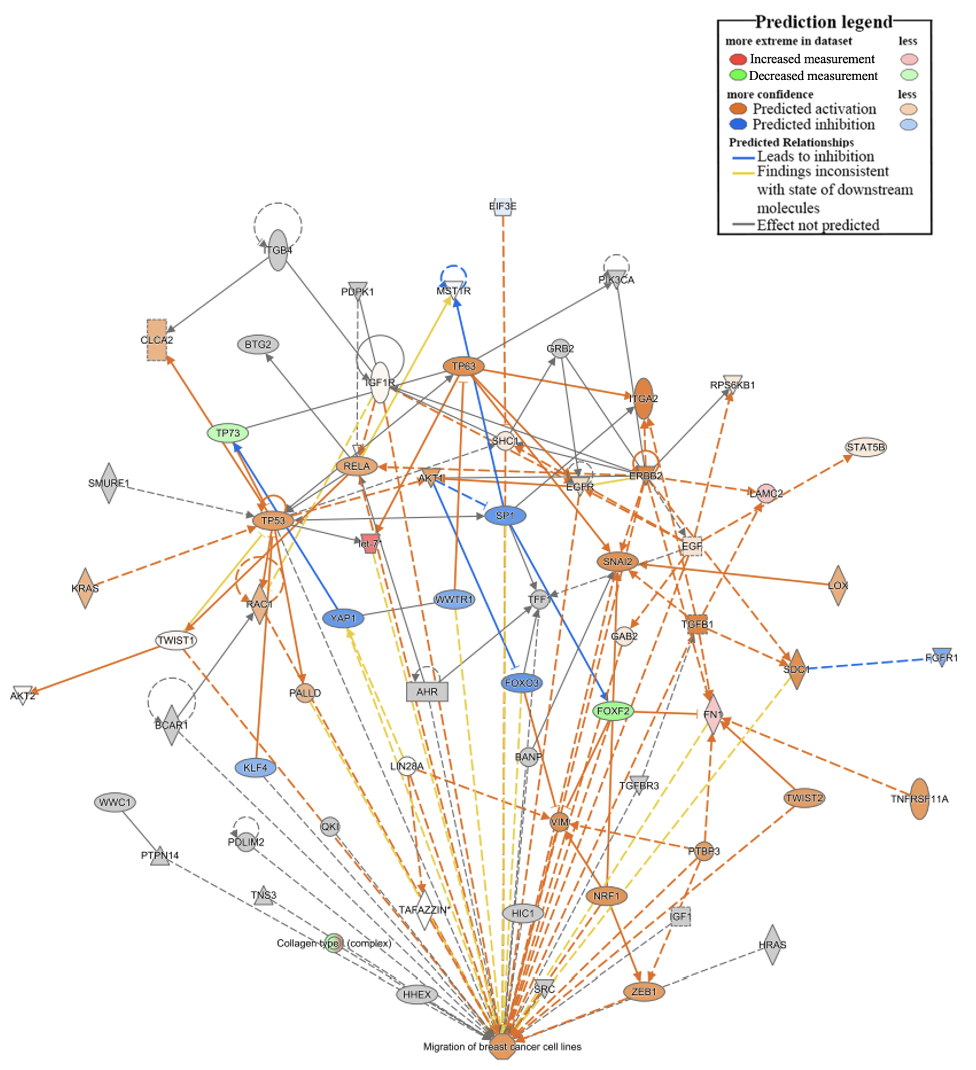


**Figure S4. KMT2B knockdown activates genes linked to the migration of BCC lines.** IPA network shows upregulation of genes related to the migration of BCCs upon KMT2B knockdown.


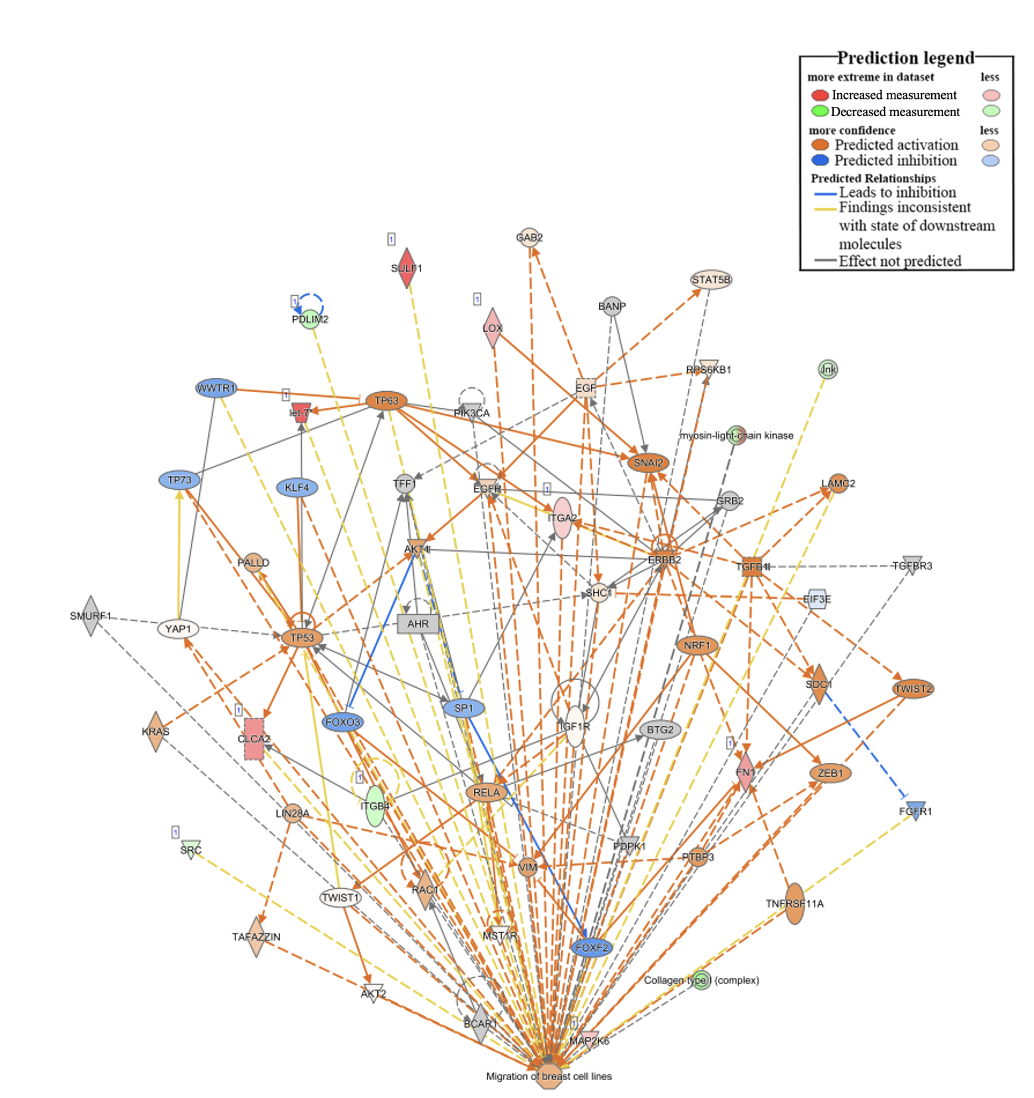


**Figure S5. KMT2D knockdown promotes the activation of genes involved in BCC migration.** IPA analysis using the RNA-seq data shows that KMT2D knockdown upregulates genes implicated in BCC migration.


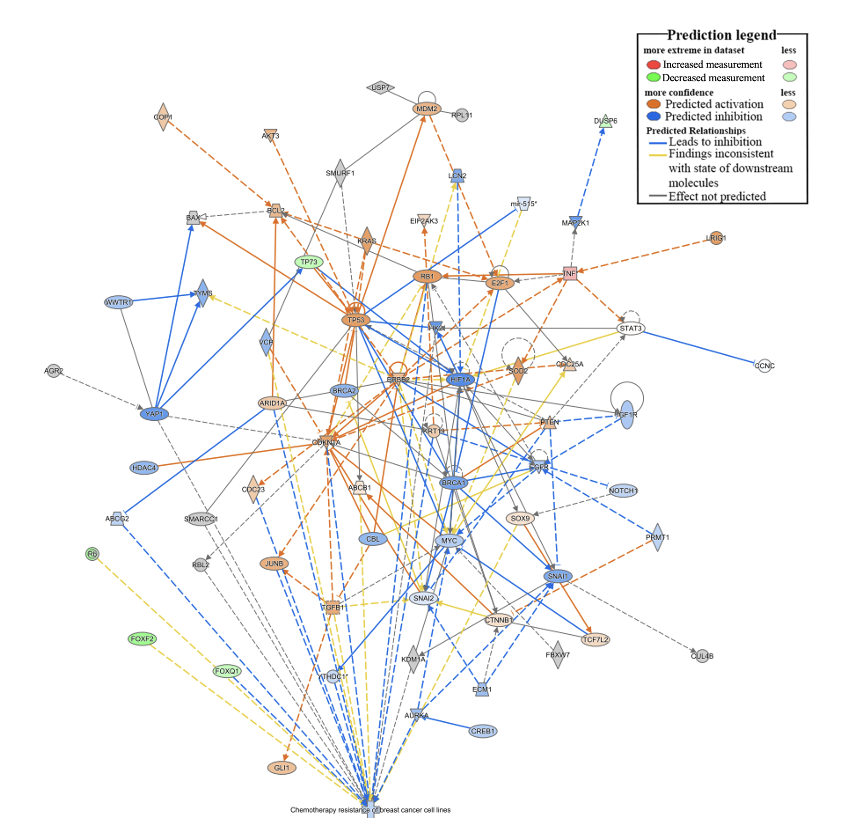


**Figure S6. KMT2B knockdown reduces chemotherapy resistance in BCCs.** *In silico* analyses illustrate that KMT2B knockdown inhibits chemotherapy resistance in BCCs.


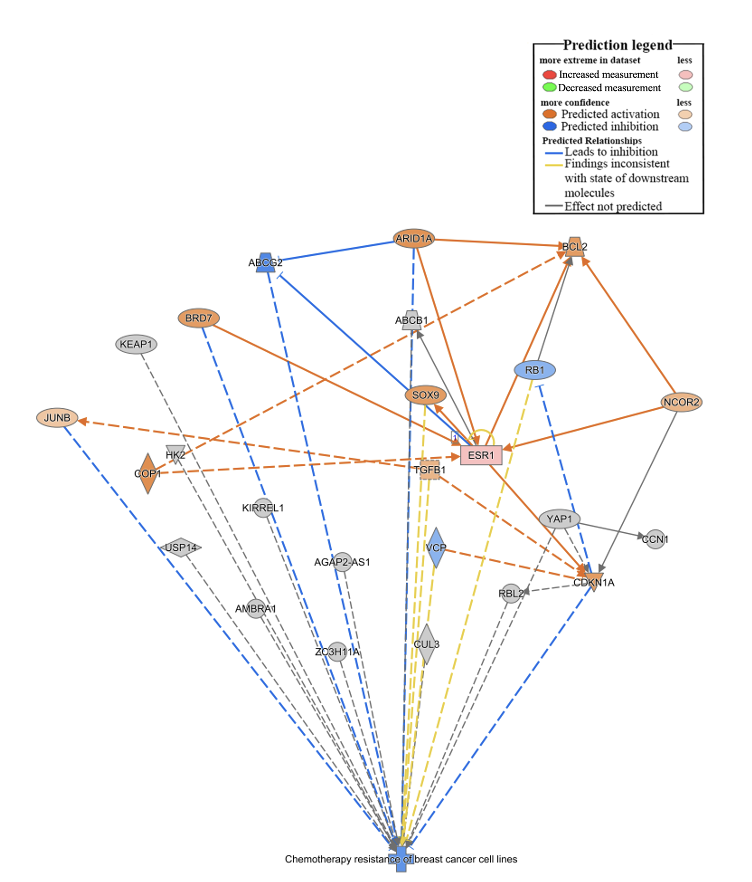


**Figure S7. KMT2D knockdown downregulates genes involved in chemotherapy resistance.** IPA network shows that KMT2D knockdown reduces chemotherapy resistance in BCCs.

**Figure S8. Orthotopic route of BC dormancy in bone marrow.** MDA-MB-231 BCCs with pOct4a-GFP (5x10^5^) were injected into the mammary fat pad of female (6 weeks) nude BALB/c. After 1 week, the mice were euthanized, and the femurs were harvested and scraped to evaluate the presence of BCCs. Shown is the fluorescence microscopy for GFP cells in the femurs of two mice. Representative image of a femur that was not injected with BCCs is shown at left. The slides were imaged with the EVOS FL AUTO 2 microscope at a magnification of 200X.
